# RSUME is implicated in tumorigenesis and metastasis of pancreatic neuroendocrine tumors

**DOI:** 10.18632/oncotarget.11081

**Published:** 2016-08-05

**Authors:** Yonghe Wu, Lucas Tedesco, Kristin Lucia, Anna M. Schlitter, Jose Monteserin Garcia, Irene Esposito, Christoph J. Auernhammer, Marily Theodoropoulou, Eduardo Arzt, Ulrich Renner, Günter K. Stalla

**Affiliations:** ^1^ Department of Clinical Neuroendocrinology, Max Planck Institute of Psychiatry, Munich, Germany; ^2^ Instituto de Investigación en Biomedicina de Buenos Aires (IBioBA)-CONICET-Partner Institute of the Max Planck Society, Buenos Aires, Argentina; ^3^ Institute of Pathology, Technical University of Munich, Munich, Germany; ^4^ Department of Internal Medicine II, University-Hospital Campus Grosshadern, Interdisciplinary Center of Neuroendocrine Tumours of the GastroEnteroPancreatic System (GEPNET-KUM), Ludwig-Maximilians-University of Munich, Munich, Germany; ^5^ Departamento de Fisiología y Biología Molecular y Celular, Facultad de Ciencias Exactas y Naturales, Universidad de Buenos Aires, Buenos Aires, Argentina; ^6^ Current address: German Cancer Research Center, Heidelberg, Germany; ^7^ Current address: Institute of Pathology, University of Düsseldorf, Düsseldorf, Germany

**Keywords:** RSUME, RWDD3, PanNETs, angiogenesis, metastasis

## Abstract

The factors triggering pancreatic neuroendocrine tumor (PanNET) progression are largely unknown. Here we investigated the role and mechanisms of the sumoylation enhancing protein RSUME in PanNET tumorigenesis. Immunohistochemical studies showed that RSUME is strongly expressed in normal human pancreas, in particular in β-cells. RSUME expression is reduced in insulinomas and is nearly absent in other types of PanNETs suggesting a role in PanNET tumorigenesis. In human pancreatic neuroendocrine BON1 cells, RSUME stimulates hypoxia-inducible factor-1α (HIF-1α) and vascular endothelial growth factor-A (VEGF-A), which are key components of tumor neovascularisation. In contrast, RSUME suppresses nuclear factor-κB (NF-κB) and its target interleukin-8 (IL-8). Correspondingly, PanNET cells with RSUME knockdown showed decreased HIF-1α activity and increased NF-κB and IL-8 production leading to a moderate reduction of VEGF-A release as reduced HIF-1α/VEGF-A production is partly compensated by NF-κB/IL-8-induced VEGF-A. Notably, RSUME stabilizes the tumor suppressor PTEN, which is frequently lost in PanNETs and whose absence is associated with metastasis formation. *In vivo* orthotopic transplantation of PanNET cells with or without RSUME expression into nude mice showed that PanNETs without RSUME have reduced PTEN expression, grow faster and form multiple liver metastases. In sum, RSUME differentially regulates key components of PanNET formation suggesting that the observed loss of RSUME in advanced PanNETs is critically involved in PanNET tumorigenesis, particularly in metastasis formation.

## INTRODUCTION

Pancreatic neuroendocrine tumors (PanNETs) are rare and represent about 1 to 3% of all neoplasias of the pancreas [1, 2]. They derive from endocrine cells producing pancreatic hormones such as insulin, glucagon, gastrin, and others, and are correspondingly designated as insulinomas, glucagonomas, gastrinomas, etc. [1, 2]. About 50 to 60% of the tumors are functionally active and are diagnosed according to their symptoms caused by the hormonal hypersecretion [2, 3]. Functionally inactive PanNETs, if not accidentally detected, are mostly recognized in advanced stages when they have formed large invasive tumors and distant metastases [1, 2, 4]. According to the WHO classification of 2010, all PanNETs are considered to be potentially malignant [1, 4]. Thus, the majority shows a malignant phenotype forming metastases in neighboring tissue or lymph nodes and almost 40–50% have hepatic metastasis at diagnosis [1, 4]. As an exception, the majority of insulinomas appear as clinically benign and only approximately 10 % of all insulinomas are malignant [1, 2, 4]. The cumulative 5-year survival rate of patients with PanNETs has been reported to be 83%, which drops to around 27% in advanced malignant stages with high Ki-67 index and the formation of distant metastases [1, 2, 4].

Complete resection of the primary tumors is the only curative therapeutic approach, however due to the formation of distant liver metastasis at diagnosis resection is only possible in a low percentage of patients [5, 6]. Palliative therapeutic options for inoperable pancreatic neuroendocrine tumors include treatment with somatostatin analogues, peptide receptor radionuclide therapy and chemotherapy using streptozocin- or temozolomide-based protocols [5, 6]. Due to the high degree of vascularization of PanNETs, the efficacy of anti-angiogenic acting drugs has also been approved [7]. Moreover, drugs targeting various receptor kinases or components of intracellular signaling pathways have been tested in recent clinical trials [7, 8]. However, the overall prognosis is still not satisfactory and better understanding of the molecular mechanisms of PanNETs development is urgent need in order to develop improved therapeutic approaches.

PanNETs are in some cases associated with hereditary genetic syndromes such as multiple endocrine neoplasia type I (MEN1), von Hippel-Lindau (VHL) syndrome, tuberous sclerosis syndrome or von Recklinghausen's disease [1, 2, 9]. Inactivating somatic mutations of several genes have also been reported in PanNETs, in particular in the tumor suppressor *MEN1*, in the chromatin interaction proteins (death-domain associated protein) DAXX/ (alpha thalassemia/mental retardation syndrome X-linked) ATRX, and in negative regulators of the PI3K-Akt-mTOR signaling cascade, e.g. phosphatase and tensin homolog (PTEN) or tuberous sclerosis 2 (TSC2) [1, 2, 10–12]. Loss of function of the tumor suppressor gene PTEN is frequently found in PanNETs and is responsible for the over-activation of the PI3K-Akt-mTOR cascade [13, 14].

PanNETs are densely vascularized and express HIF-1α and VEGF-A [7, 15]. In contrast to many types of tumors, in which loss of differentiation goes along with an increase of microvessel density, less well-differentiated PanNETs are paradoxically less vascularized probably due to an impaired expression of the HIF-1/VEGF-A pathway [15]. VEGF-A is regulated by HIF-1, which plays a key role in tumor neovascularization and is composed of the constitutively expressed HIF-1β and the oxygen-sensitive HIF-1α subunit [16]. The latter is continuously produced but subsequently degraded by ubiquitinylation under normoxic conditions. Under hypoxia, as it occurs in expanding tumors, this process is rapidly inhibited leading to the production of active an HIF-1 complex, which then induces the production of multiple angiogenic factors including VEGF-A [16].

We have previously shown that RSUME, the product of the RWDD3 gene, is a RWD containing protein that stabilizes and enhances HIF-1α [17–19]. RSUME also stabilizes I-κBα, the natural inhibitor of NF-κB, thereby inhibiting NF-κB [17, 19]. The latter is overexpressed in many types of tumors, including pancreatic adenocarcinomas, where it plays a key role in mediating inflammatory signals and triggering proliferation, angiogenesis and metastasis [20]. One of the NF-κB targets, interleukin-8 (IL-8), is strongly expressed in PanNETs, suggesting NF-κB over-activation [21]. Since pancreas strongly expresses RSUME [17], which is involved in the regulation of key molecules and processes influencing PanNETs, the aim of the study was to explore the expression and role of RSUME in PanNET tumorigenesis. We found that loss of RSUME is a characteristic of PanNETs and may contribute to tumor angiogenesis and metastasis.

## RESULTS

### RSUME is down-regulated in human pancreatic neuroendocrine tumors

**Figure 1:**
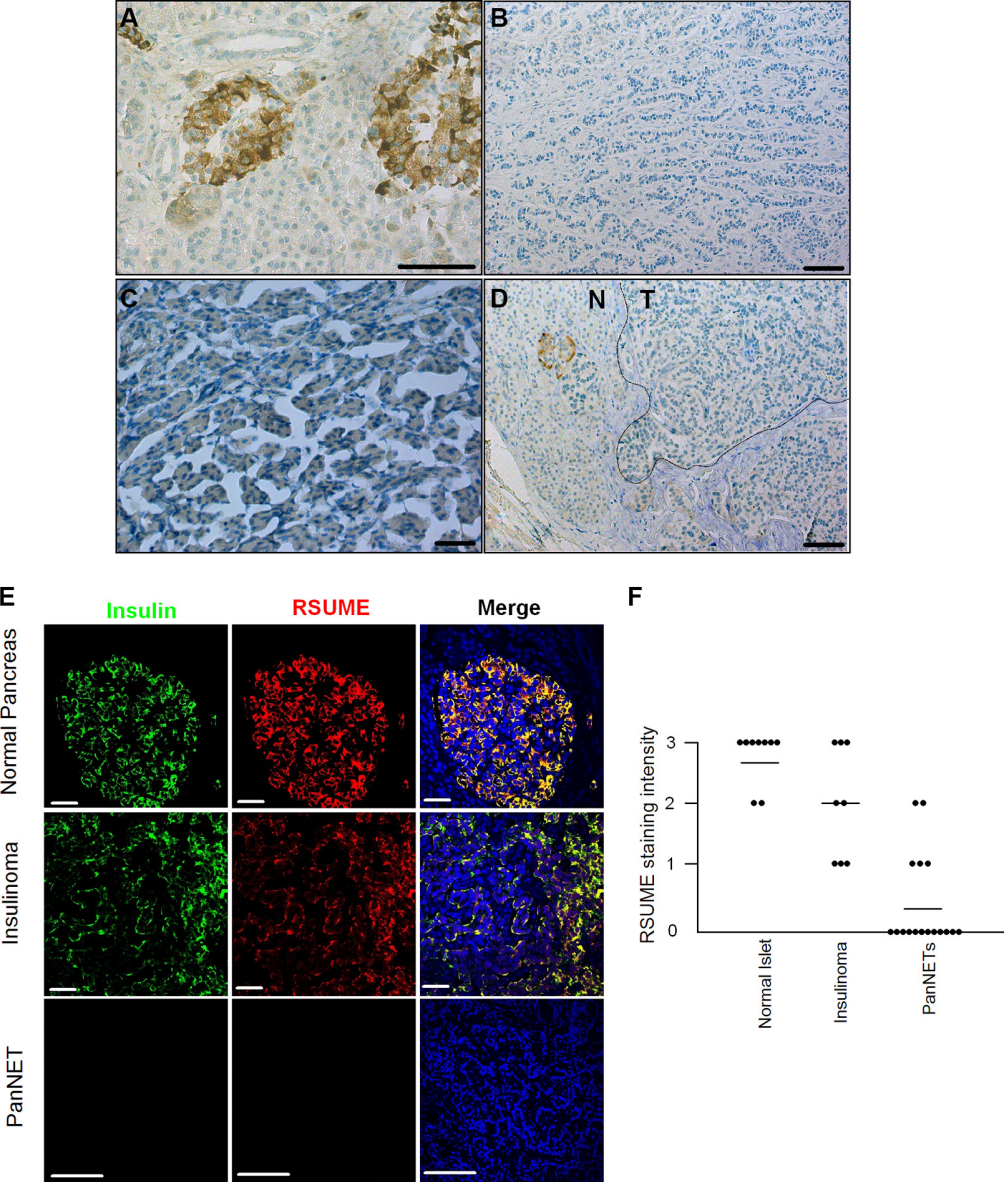
RSUME expression is decreased in human pancreatic neuroendocrine tumors Immunohistochemistry staining of RSUME in resected normal pancreas (**A**), PanNETs (**B**, Grade 2), insulinoma (**C**) and PanNET with a non-malignant normal region (**D**, Grade 1). (**E**) Co-staining of Insulin (green) and RSUME (red) in normal pancreas, insulinoma, and other types of PanNETs. Images are representative of three experiments with similar results. Scale bar 50 μm. (**F**) Summary of RSUME expression in normal pancreas, insulinoma and other types of PanNETs. The intensity of the staining was classified as negative (0), weakly (1+), medium (2+) and strongly positive (3+). All samples from this study were assessed by two different raters who were blinded to each other. See Table 1 for detailed patient information.

Immunohistochemical studies showed strong RSUME expression in the insulin-positive cells of the Langerhans islets in the normal pancreas (*n* = 9) (Figure 1A, 1E), in which somatostatin-positive cells also expressed RSUME (Supplementary Figure 1). Moderate expression of RSUME was also found in exocrine acinar cells whereas RSUME was absent in ductal cells (Figure 1B–1E). Among 24 islet 1-positive PanNETs [22] investigated (11 G1 and 13 G2 tumors; Table 1, Supplementary Figure 2), scattered cytoplasmatic RSUME immunopositivity was observed in insulinomas (*n* = 7; Figure 1C, 1E) whereas RSUME was absent in the vast majority of the other PanNETs including 4 somatostatin expressing tumors (Figure 1B, 1D, 1E; Supplementary Figure 1). Thus, in comparison to the normal pancreas, RSUME expression is decreased in PanNETs (Figure 1F).

**Table 1:** Clinicopathological features of PanNET patients

	Diagnosis	Grading	Proliferation index	Hormone Expression	RSUME expression	PTEN level	PTEN subloc.	Islet1
1	PanNET	G1	3%	Not tested	−	−		+++
2	PanNET PanNET,	G1	< 1%	Not tested	−	++	N + C	+++
3	lymphnode metastasis	G2	15%	No expression	−	++	C	+++
4	Insulinoma	G1	3%	Insulin+	++	++	C	+++
5	PanNET	G2	NA	Not tested	−			+++
6	PanNET	G2	NA	Insulin neg.	++	+	C	+++
7	PanNET	G1	1%	Not tested	−	−		+++
8	PanNET	G2	4%	Not tested Serotonin+,	−	−		+++
9	PanNET	G2	NA	Somatostatin+ Gastrin+	−	+	C	+++
10	PanNET	G2	3%	Not tested	−	+++	C	+
11	PanNET	G2	10%	Somatostatin+	−	−		+
12	PanNET	G2	7%	Insulin neg.	+	−		++
13	PanNET	G1	< 2%	Insulin neg.	−	++	C	++
14	PanNET	G2	15%	No expression	−	−		+++
15	PanNET PanNET,	G2	3%	Somatostatin+	−	−		+++
16	lymphnode metastasis PanNET,	G1	1%	Glucagon+	−	++	C	++
17	lymphnode metastasis	G1	< 1%	Somatostatin+ VIP	−	+	N + C	+++
18	PanNET	G2	4%	Serotonin+, Insulin neg.	++	−		+++
19	Insulinoma	G2	NA	Insulin+	+	−		+++
20	Insulinoma Insulinoma,	G1	NA	Insulin+	+++	+	C	−
21	liver metastasis	G2	5%	Insulin+	+	−		+++
22	Insulinoma	G1	< 2%	Insulin+	+++	−		+++
23	Insulinoma	G2	3%	Insulin+	+	++	C	+++
24	Insulinoma	G1	NA	Insulin+	+++	++	C	+++

PanNET: pancreatic neuroendocrine tumor; NA: not available; N: Nuclear staining; C: cytoplasm staining; subloc: sublocalization.

### RSUME regulates angiogenesis through the HIF-1α/VEGF pathway

**Figure 2:**
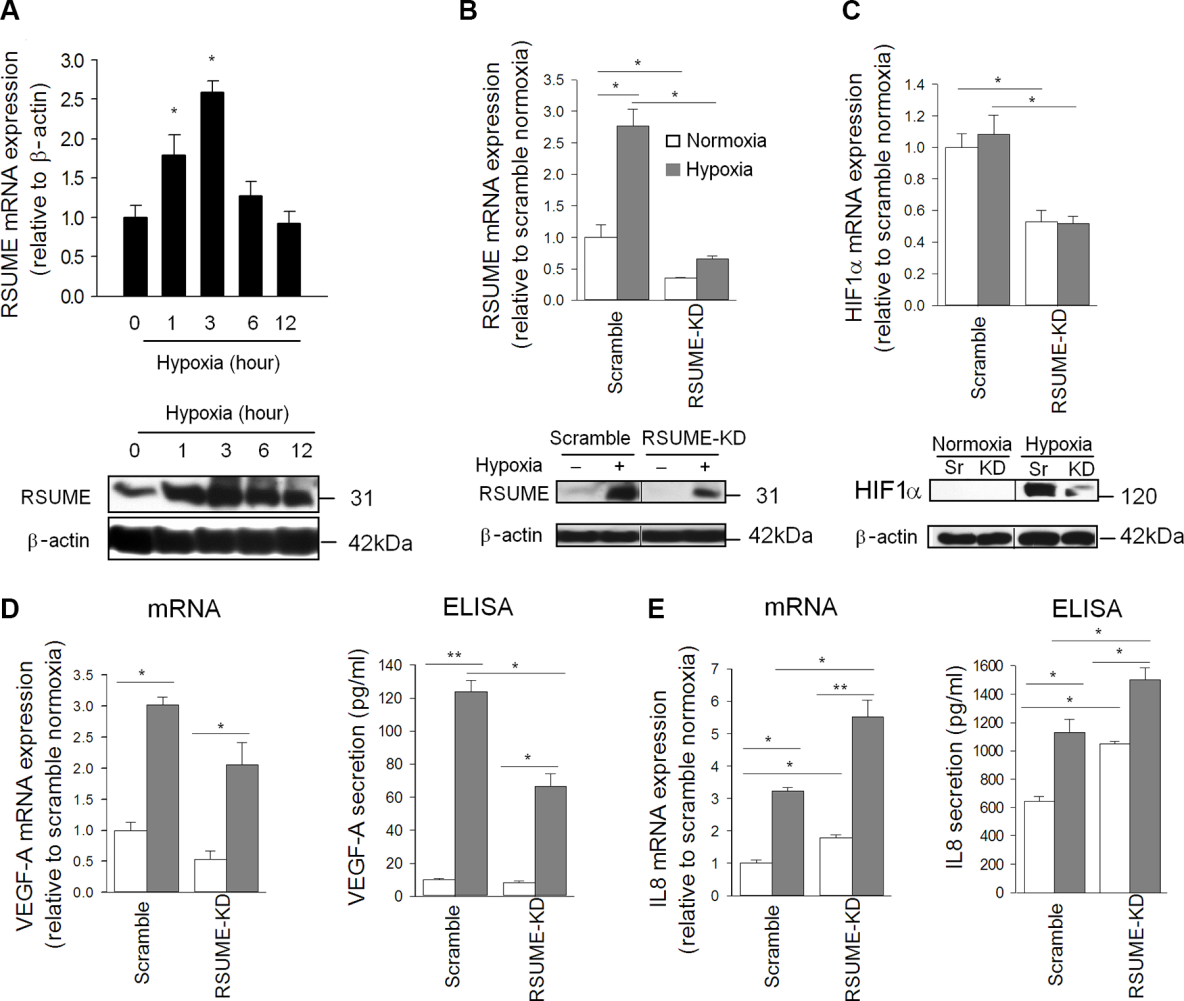
Influence of RSUME on HIF-1α, VEGF-A and IL-8 production RSUME mRNA and protein level (**A**) were stimulated during hypoxia (1% O_2_) for the indicated time points in BON1 cells. As expected RSUME mRNA and protein was down-regulated and less sensitive to hypoxia in BON1 cells with RSUME knock-down (BON1^RSUME-KD^) compared to scramble siRNA trasfected cells (BON1^Scramble^) (**B**). HIF-1a mRNA and in particular hypoxia-induced HIF-1a protein production was strongly impaired in BON1^RSUME-KD^ cells (**C**). Normoxic and hypoxic mRNA synthesis and secretion of VEGF-A was suppressed in BON1^RSUME-KD^ cells (**D**) whereas IL-8 mRNA and protein production was enhanced. (**E**). All experiments were performed three times and in B to E treatment time was 3 h for mRNA expression and 12 h for protein production studies, respectively. Results are expressed as mean ± SEM of triplicates for mRNA and quadruplicate for ELISA. *P < 0.05, **P < 0.01.

In neuroendocrine pancreatic BON1 cells, RSUME mRNA and protein was enhanced by hypoxia (1% O_2_; Figure 2A) or hypoxia mimicking conditions (CoCl_2_ treatment, Supplementary Figure 3) through RSUME/HIF-1 interaction (Supplementary Figure 4) as already shown in other cell types [17, 23]. Knocking down RSUME (BON1^RSUME-KD^) decreased basal and hypoxia-induced RSUME and HIF-1α mRNA and protein levels (Figure 2B, 2C), confirming the importance of RSUME in HIF-1α regulation. In comparison to the strong suppression of moderately inhibited basal and hypoxia-induced mRNA levels and secretion of VEGF-A (Figure 2D) suggesting the induction of a compensatory mechanism to preserve VEGF-A production. In neuroendocrine pancreatic QGP1 cells, RSUME overexpression induced HIF-1a expression during hypoxia confirming the impact of RSUME on HIF-1α regulation (Supplementary Figure 5).

In HIF-1α deficient colon cancer cells, VEGF-A production is preserved by the pro-angiogenic cytokine IL-8 [24]. We found expression of IL-8 and its receptor CXCR2 in BON1 cells and in the human neuroendocrine carcinoma QGP1 cell line (Supplementary Figure 6). The CXCR2 inhibitor SB225002 significantly reduced basal and hypoxia-induced VEGF-A secretion (Supplementary Figure 7). RSUME knockdown increased IL-8 transcription and secretion, which was further induced by hypoxia (Figure 2E). Increased levels of IL-8 can stimulate VEGF-A, which may explain that the loss of RSUME in PanNET cells has limited inhibitory effects on VEGF-A secretion despite strongly decreased HIF-1α.

### RSUME negatively regulates NF-κB activity by enhancing IκBα sumoylation in PanNETs

**Figure 3:**
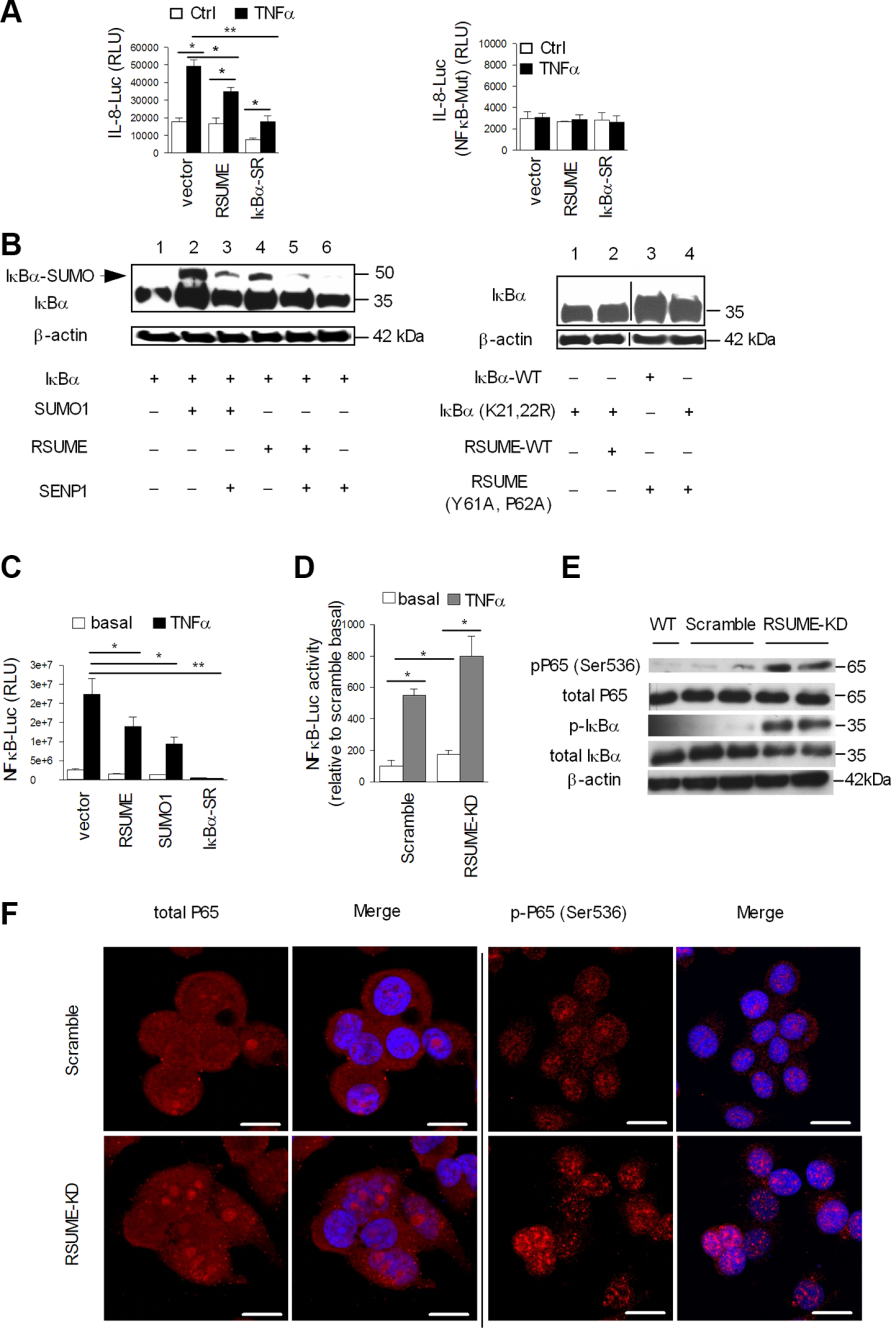
RSUME negatively regulates NF-κB activity by enhancing sumoylation of IκBα BON1 cells were transfected with IL-8-LUC (**A**, left) or IL-8 (NF-κB-mut)-LUC (A, right) reporter vector, RSUME or IκBα super repressor (I-κBα-SR) and β-gal plasmid. After 24 h, cells were stimulated with 10 ng/ml TNF-α for 6 h, and LUC activity was measured in the cell extracts. (**B**) BON1 cells were co-transfected with I-κBα, I-κBα mutated at the SUMO1 conjunction target sites lysine 21 and 22 (K21, 22R), and the indicated expression vectors (including the SUMO1/sentrin specific peptidase 1 -SENP1), to analyze I-κBα sumoylation status in BON1 cells. After 24 h, cell extracts were subjected to WB with anti-I-κBα antibody. β-actin was used as the loading control. (**C**) BON1 cells were transfected with NF-κB-LUC reporter vector, RSUME, HA-SUMO1 or I-κBα expression vectors. After 24 h, cells were stimulated with 10 ng/ml TNF-α for 6 h, and LUC activity was measured. (**D**) NF-κB-Luc activity in BON1^RSUME-KD^ and BON1^Scramble^ cells was measured with or without TNF-α (10 ng/ml, 6 h). All values were normalized to β-gal activity (mean ± SEM of 3 different experiments, *t* test). *P < 0.05, **P < 0.01 (**E**) Western blot experiments were performed with cell extracts were RSUME was knockdown and with Immunoblotting for NF-κB subunits (p65, p-p65(Ser536), I-κBα and pI-κBα (Ser32/36) in BON wild type, BON1^RSUME-KD^ and BON1^Scramble^ cells lysates. β-actin was used as the loading control. (**F**) Immunofluorescence for total and phosphor-p65 (Ser536) in BON1^RSUME-KD^ and BON1^Scramble^ cells. Antibodies used were indicated above each image with corresponding colors. DAPI staining was used to visualize the nucleus. Scale bars are equal to 10 μm. Each image is representative of three independent experiments with similar results.

IL-8 expression is stimulated by NF-κB [25]. RSUME overexpression inhibited TNFα-induced IL-8 promoter activity and co-transfection with the I-κBα super repressor (I-κBα-SR) significantly attenuated this effect (Figure 3A, left). All these effects were completely abolished when the NF-κB binding site of the IL-8 promoter was mutated, which further demonstrates that RSUME inhibits IL-8 activity through NF-κB in BON1 cells (Figure 3A, right). RSUME overexpression increased I-κBα sumoylation, an effect which was comparable to that of SUMO1 (Figure 3B, left, upper band, lanes 2 and 4). This effect was abolished when I-κBα was mutated at the SUMO1 conjunction target sites lysines 21 and 22 (Figure 3B, right, lane 1 and 2) [26] or overexpression of the RSUME-Mut (Y61A, P62A) where the highly conserved YPXXXP motif in the RWD domain of RSUME was mutated (Figure 3B, right, lane 3 and 4) [17, 22]. Co-transfection with the SUMO1/sentrin specific peptidase 1 (SENP1), attenuated sumoylated I-κBα (Figure 3B, left, lanes 3, 5, 6) demonstrating that RSUME specifically affects I-κBα sumoylation. RSUME suppressed basal and TNFα-induced NF-κB transcriptional activity similar to SUMO1, and this effect was abolished by the I-κBα super-repressor (Figure 3C). In contrast, RSUME knockdown increased both basal and TNFα-induced NF-κB transcriptional activity (Figure 3D), further demonstrating the repressive role of RSUME on NF-κB activity in BON1 cells.

Western blot experiments were performed with cell extracts using antibodies against key components of the NF-κB pathway, including total and phosphorylated p65/RelA and I-κBα. RSUME knockdown increased phosphorylated I-κBα (Ser32/36) and p65/RelA (Ser536) levels (Figure 3E), indicating NF-κB activation. Furthermore, loss of RSUME is accompanied by increased p65/RelA (Ser536) nuclear translocation (Figure 3F), which is indicative of activated NF-κB.

### RSUME enhances SUMO-directed PTEN sumoylation

**Figure 4:**
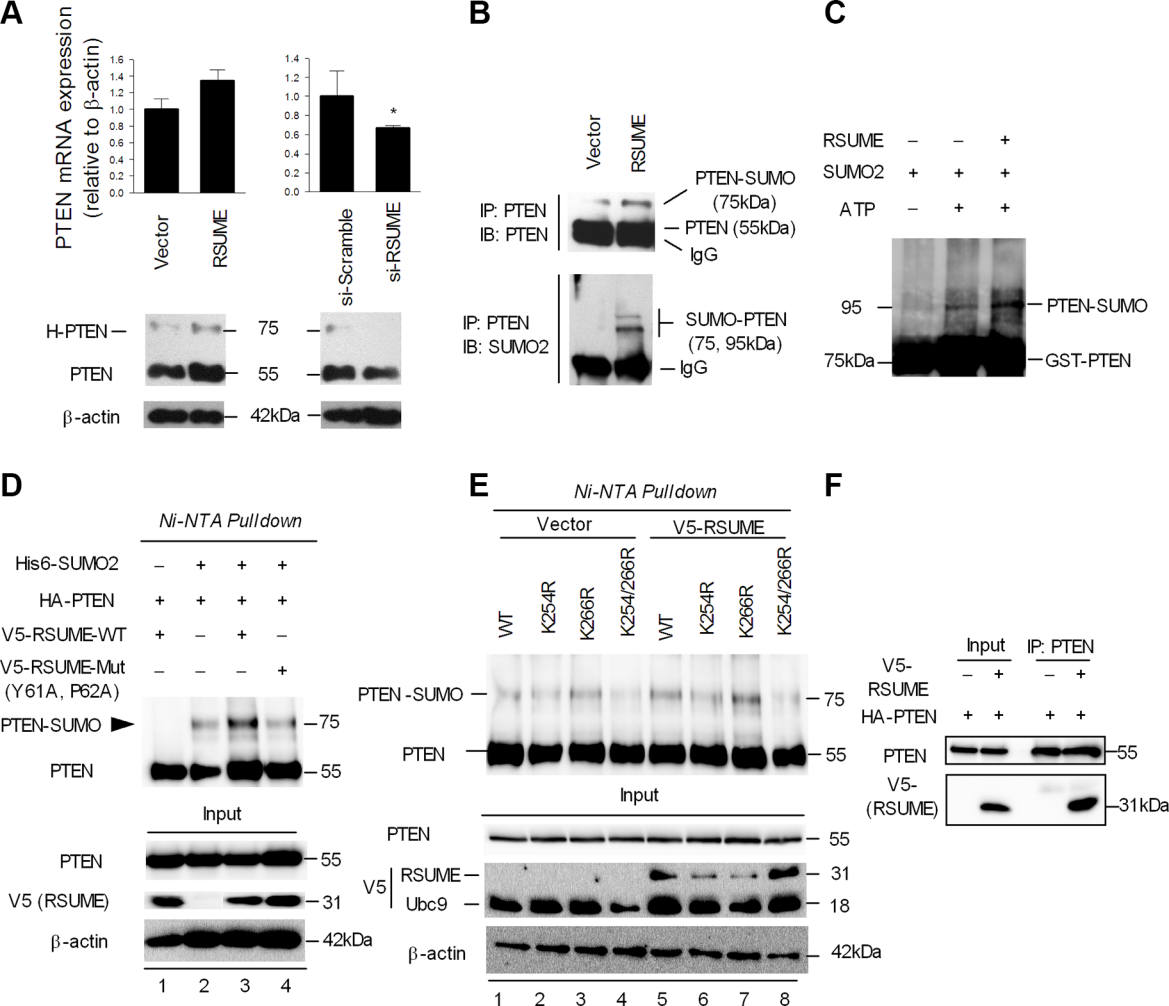
RSUME enhances SUMO-induced PTEN sumoylation (**A**) PTEN mRNA expression was measured in RSUME overexpressing (right) and *RSUME* silenced (left) BON1 cells. β-actin was used for normalization. Immunoblot for PTEN was analyzed in *RSUME* silenced (left) and RSUME overexpressing (right) BON1 cells. β-actin was used for normalization. High-migration PTEN (H-PTEN). (**B**) Immunoprecipitation was performed with a PTEN antibody in BON1 cells followed by western blot using antibodies against PTEN and SUMO2. (**C**) GST-PTEN was incubated using an *in vitro* sumoylation assay mixture containing SUMO2 with or without RSUME. After incubation, reactions were stopped by adding loading buffer and subsequently an immunoblot was performed with anti-PTEN antibody. (**D**) COS7 cells were co-transfected with HA-PTEN, wild type V5-RSUME or the mutated V5-RSUME (Y61A, P62A), His6-SUMO2 and V5-Ubc9. 48 h post-transfection, cells were lysed, purified by Ni-NTA and immunoblotted with indicated antibodies. (**E**) COS7 cells were co-transfected with HA-PTEN expression or sumoylation-deficient PTEN mutants (K254R, K266R, K254/266R), His6-SUMO2 and V5-RSUME plasmids. 48 h post-transfection, cells were lysed, Ni-NTA purified and immunoblotted with the indicated antibodies. (**F**) COS7 cells were transfected with HA-PTEN with or without V5-RSUME. 48 h post-transfection, cells were lysed with RIPA buffer (10% for Input), precipitated with PTEN antibody and subsequently immunoblot was performed with antibodies indicated. For all experiments, one representative experiment from two independent experiments with similar results is shown.

NF-κB was previously shown to suppress PTEN [27], which is reduced in 16 of 24 of the PanNETs studied (Table 1). Since RSUME negatively regulates NF-κB activity, we investigated its role on PTEN transcription. RSUME overexpression in BON1 cells increased PTEN mRNA and protein levels and the opposite was observed after RSUME knockdown (Figure 4A). In QGP1 cells RSUME overexpression also enhanced PTEN expression (Supplementary Figure 5). The functional significance of RSUME's effect on PTEN is evidenced by the increased Akt phosphorylation in RSUME knockdown cells (Supplementary Figure 8). In addition, we observed a high migration band (~75 kDa, H-PTEN), which is regulated by RSUME, indicating posttranslational modification (Supplementary Figure 8). RSUME knockdown decreased total and 75kDa H-PTEN: total PTEN ratio (13.13 ± 4.77% vs. 5.97 ± 1.95%, Supplementary Figure 8). We confirmed that the higher migration band at ~75 kDa is SUMO-PTEN by co-immunoprecipitation with SUMO2 (Figure 4B). An *in vitro* sumoylation assay revealed that the band detected between 75 and 100 kDa (~26 kDa for GST-tag) was SUMO2-modified PTEN, and this signal was further enhanced by RSUME (Figure 4C). A SUMO conjugation assay confirmed the increase in 75 kDa sumoylated PTEN levels in RSUME overexpressing cells (Figure 4D, lane 3). RSUME also enhanced SUMO1-mediated PTEN sumoylation, but to a lesser extent (Supplementary Figure 9). To further clarify the mechanisms through which RSUME regulates PTEN we used COS7 cells as we assume that the effects of RSUME on PTEN may be similar in each RSUME expressing cell type. RSUME-Mut (Y61A, P62A) failed to stimulate PTEN modification by SUMO2 (Figure 4D, lane 4). *In silico* prediction (SUMOplot^™^ Analysis Program) and previous reports [28, 29] revealed two major sumoylation conjugation sites (lysine 254 and lysine 266) in PTEN. Sumoylation deficient PTEN mutants (K254R, K266R, K254/266R) displayed variable sumoylation (Figure 4E, lanes 2–4). The expected ~75 kDa was observed in Ni-NTA purified proteins when cells were co-transfected with wild type PTEN (WT-PTEN) and His-SUMO2. The single K254R mutant showed reduced sumoylation (Figure 4E, lane 2 vs. lane 1), which was further reduced in the double K254/266R mutant (lane 4). In contrast, the single K266R mutant (lane 3) had a minor effect on sumoylation, but exerts a synergistic effect with the K254 site. RSUME overexpression strongly enhanced SUMO2-induced sumoylation in the WT-PTEN construct (lane 5), but not in the double K254/266R mutant (lane 8). Co-immunoprecipitation revealed a physical association between RSUME and PTEN in cells (Figure 4F).

### RSUME increases PTEN stability by reducing its ubiquitination

**Figure 5:**
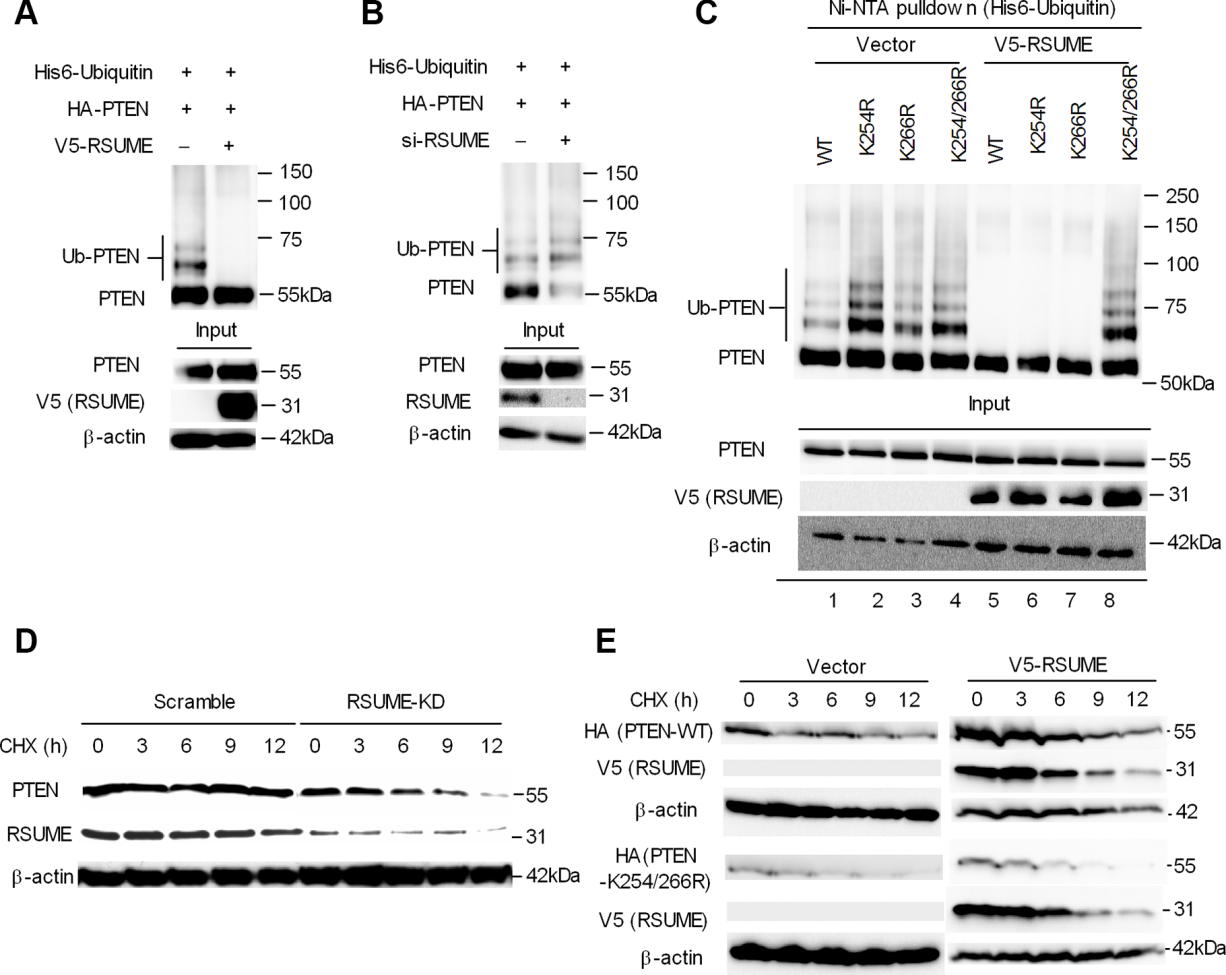
RSUME inhibits PTEN ubiquitination and increases protein stability (**A**) COS7 cells were transfected with HA-PTEN, His6-ubiquitin and V5-RSUME or a backbone construct pCEFL. 48 h post-transfection, cells were incubated with 5 μM MG132 or vehicle for 6 h. His-ubiquitinated (His-Ub) proteins were isolated from denatured whole-cell extracts and pulled down by nickel beads. Purified proteins and input samples (whole-cell extracts) were analyzed by western blotting with anti-PTEN and anti-V5 (RSUME) antibodies. Signal in His-Ub pull-down lanes corresponds to ubiquitinated PTEN. b-actin was used as a loading control. (**B**) COS7 cells were transfected with HA-PTEN, His6-ubiquitin and 10 μM siRSUME. 48 h posttransfection, cells were incubated with MG132, purified and immunoblotted with anti-PTEN and anti-RSUME. β-actin was used as a loading control. (**C**) COS7 cells were transfected with HA-PTEN or different mutants (K254R, K266R, K254/266R), V5-RSUME and His6-Ubiquitin. 48 h post-transfection, cells were incubated with MG132, purified and immunoblotted with anti-PTEN and anti-RSUME. One representative experiment from two independent experiments with similar results is shown. (**D**) BON1^RSUME-KD^ and BON1^Scramble^ cells were treated with cycloheximide (CHX) for the time indicated. Cell lysates were collected and subjected to immunoblot using antibodies against PTEN and RSUME. β-actin was used as the loading control. (**E**) COS7 cells were co-transfected with wild type or sumoylation-deficient PTEN mutant (K254/266R) with or without V5-RSUME. 48 h post-transfection, cells were treated with cycloheximide for the indicated time points and immunoblotted with the indicated antibodies. β-actin was used as the loading control.

We next investigated if the RSUME-induced PTEN sumoylation modulates PTEN protein stability. RSUME overexpression strongly reduced PTEN ubiquitination levels in COS7 cells, indicative of enhanced protein stability (Figure 5A). The inhibitory role of RSUME on PTEN ubiquitination was further confirmed by silencing RSUME in COS7 cells (Figure 5B). PTEN-K254R and the double mutant K254/266R displayed increased ubiquitination compared to wild type, while the single K266R mutation had a minor effect on PTEN ubiquitination. RSUME overexpression inhibited PTEN ubiquitination in the wild type as well as single mutants (K254R and K266R), suggesting that a single mutation at the sumoylation acceptor site is not sufficient to prevent RSUME-induced PTEN ubiquitination inhibition. When both PTEN sumoylation acceptor sites were mutated, RSUME overexpression had no inhibitory effect on PTEN ubiquitination. These results indicate that RSUME inhibits PTEN ubiquitination and that both sumoylation acceptor sites (lysine 254 and lysine 266) are indispensable for sustaining this effect (Figure 5C).

RSUME knockdown in BON1 cells showed decreased PTEN half-life during cycloheximide (CHX) treatment while it had little effect in the scramble transfected control cells (Figure 5D). Similarly, the sumoylation deficient PTEN-K254/266R displayed decreased protein half-life compared to WT-PTEN indicating the critical role of sumoylation for PTEN protein stabilization. RSUME overexpression significantly increased WT-PTEN protein stability, but it had little effect on protein stability of the double mutant K254/266R (Figure 5E).

### RSUME increases PTEN nuclear accumulation

**Figure 6:**
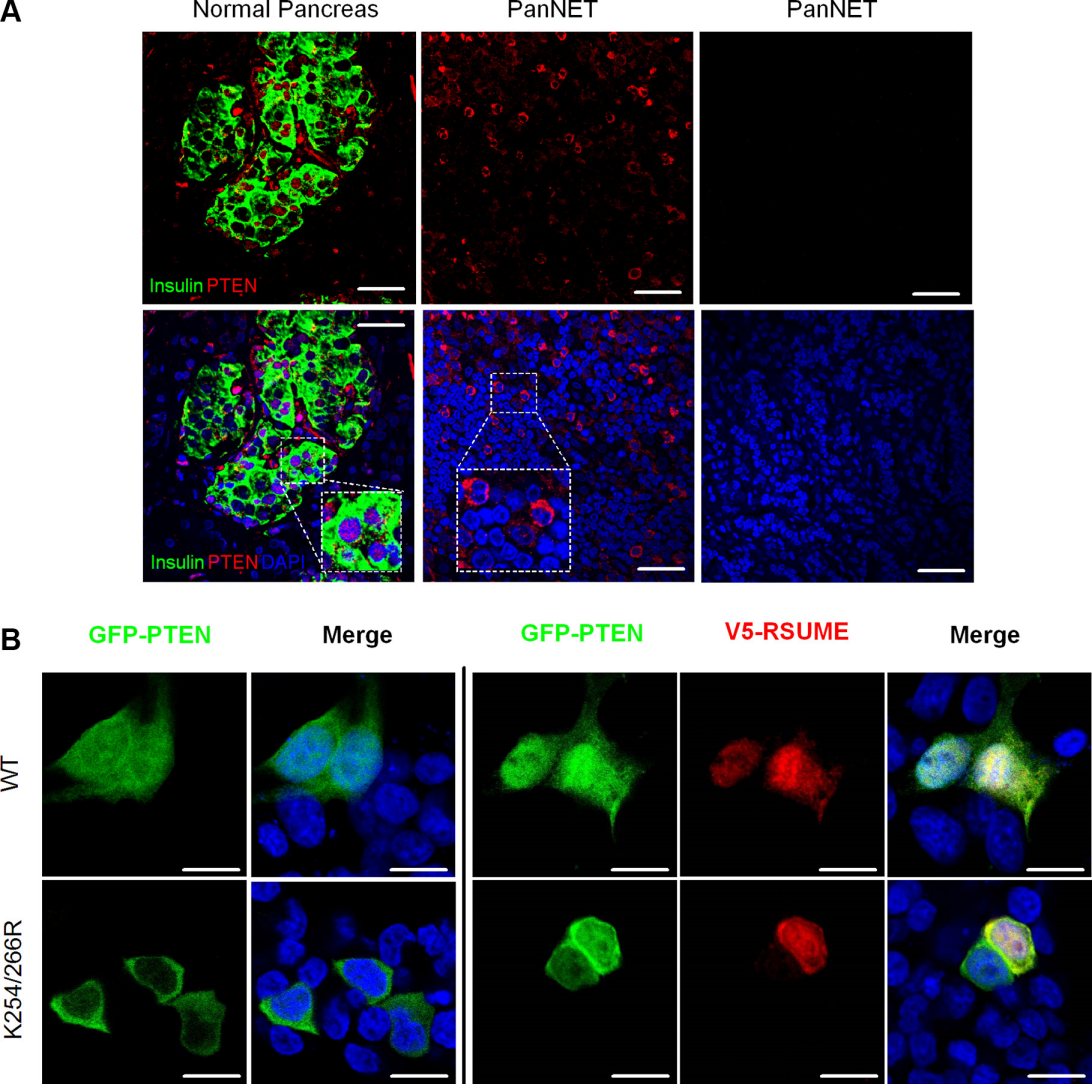
RSUME influences PTEN localization in normal and tumoral pancreatic cells (**A**) Immunofluorescence studies showed that in the normal pancreas, PTEN (red) is predominantly localized in the nuclei of insulin-producing cells (green) (A, left) whereas in PanNETs PTEN is present in the cytoplasm of tumor cells (A, middle) or completely absent (A, right). PTEN showed both nuclear and cytoplasmic expression in BON1 cells transfected with GFP-PTEN (green) whereas the PTEN sumoylation deficient double mutant (K254/266R) (green) is localized only in the cytoplasm (**B**, left). Over-expression of RSUME (red) in GFP- or K254/266R-PTEN expressing BON1 cells strongly increased nuclear PTEN (green) expression in cells with GFP-PTEN whereas PTEN remained in the cytoplasm of cells expressing the K254/266R mutant (B, right). Cell nuclei were visualized using DAPI (blue). Scale bar: 10 μm.

PTEN nuclear localization is pivotal for its anti-tumorigenic activity and is regulated by sumoylation [28, 29]. PTEN displayed differential sublocalization between the normal pancreas and PanNETs (Figure 6A, Table 1), similar to previous observation [30]. In the normal pancreas, PTEN had strong nuclear and cytoplasmic staining in insulin-producing islet cells (Figure 6A, left), whereas in PanNET samples, PTEN showed either loss of expression (Figure 6A, right) or predominantly cytoplasmic staining (Figure 6A, middle). Wild type GFP-PTEN was equally distributed in both the nucleus and cytoplasm while the PTEN mutants (K254R, K266R, K254/266R) showed predominantly cytoplasmic localization (Figure 6B, left). RSUME overexpression (red) dramatically increased nuclear PTEN accumulation (Figure 6B, right). Similar results were obtained in HEK293 and PTEN-null prostate PC3 cells (Supplementary Figure 10). RSUME overexpression had only moderate effects on the nuclear translocation of the single PTEN-K254R and PTEN-K266R mutants but clearly reduced nuclear translocation double PTEN-K254/266R mutant, which remained predominantly in the cytoplasm (Figure 6B, Supplementary Figure 10). In sum, RSUME facilitates PTEN nuclear accumulation by modifying its sumoylation status.

### RSUME knockdown alters tumorigenic factors and favors metastasis formation in an orthotopic tumor model

**Figure 7:**
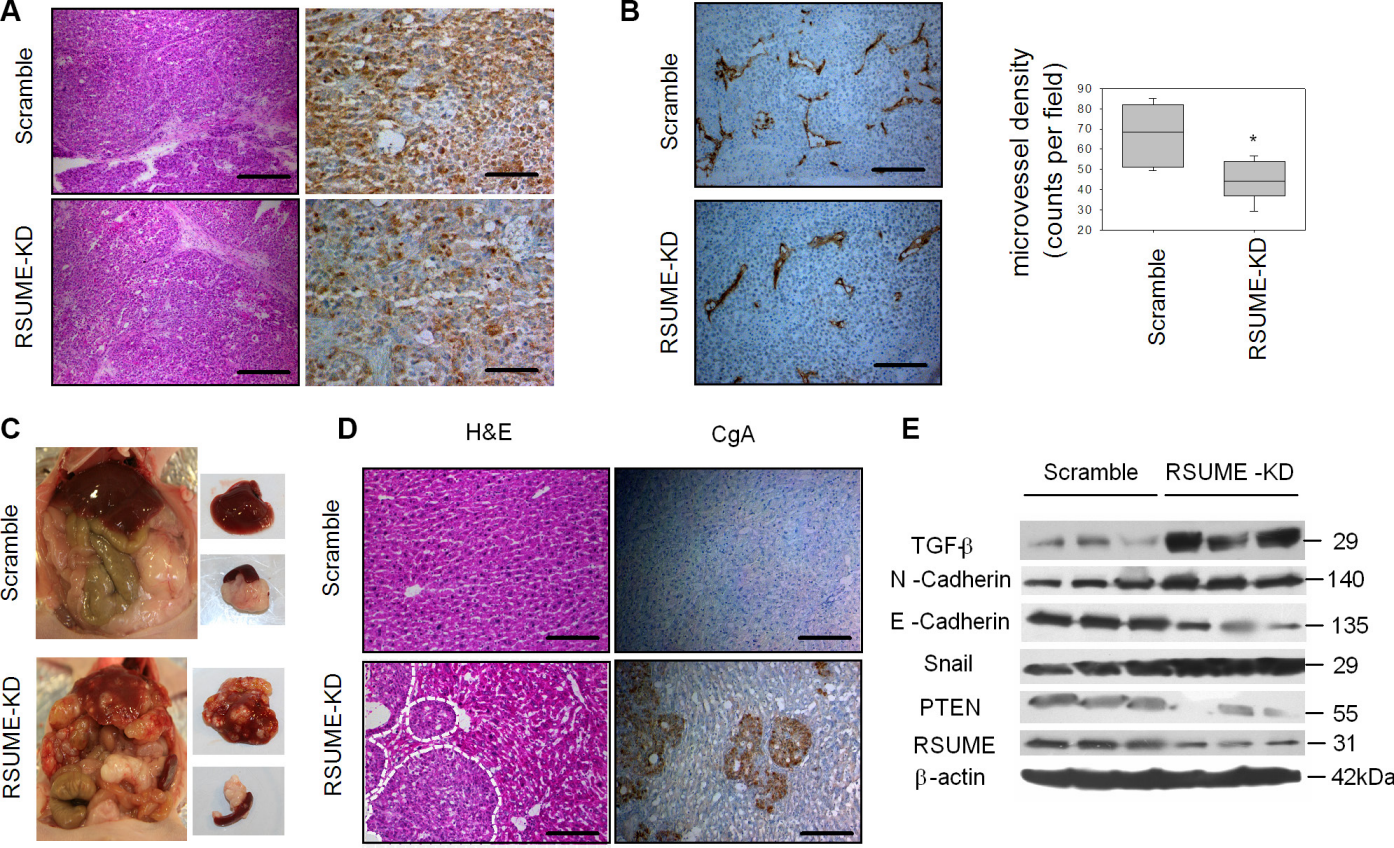
Role of RSUME in an orthotopic neuroendocrine pancreatic tumor model Orthotopic tumors from BON1 cells without (scramble) or with RSUME knockdown (RSUME-KD) were generated by injecting the cells into the pancreas of athymic nude mice. In comparison to scramble tumors, RSUME-KD tumors showed reduced chromogranin A staining (brown) indicative of a loss of neuroendocrine differentiation (**A**). RSUME-KD tumors are significantly less vascularised (CD31 staining) than scramble tumors (**B**) but show strongly enhanced spread of metastases into the liver as shown morphologically (**C**) and immunohistochemically by detecting CgA-positive neuroendocrine tumor tissue in the nude mouse liver (**D**). Tumors with RSUME-KD show reduced PTEN expression and signs of epithelial-mesenchymal-transition (EMT) such as enhanced TGF-β, N-Cadherin and Snail protein levels as well as reduced E-Cadherin protein expression (**E**) indicating that loss of RSUME promotes EMT in PanNETs. Microvessel density in the right panel in B was determined by counting of the number of vessels in one field under 100× magnification. Results were obtained from 6 independent pictures for each condition and are expressed as mean ± SEM. *P < 0.05 vs. scramble tumors. Scale bar 100 μm.

Our data show that loss of RSUME upregulates NF-κB and downregulates PTEN, and both these effects may contribute to enhanced PanNET tumorigenesis. We made orthotopic xenografts by implanting human pancreatic BON1 PanNET (scramble and RSUME-KD) cells into the pancreas of nude mice, which was proven to be a successful PanNET model [31]. All animals developed tumors in the pancreas by 9 weeks and the tumors from the RSUME-KD group showed much higher weight compared to scramble controls (1102.5 ± 78.5 versus 786 ± 56.2 mg, Supplementary Figure 12). BON1^RSUME-KD^ tumor samples exhibited significantly higher Ki67 proliferation index (12.70 ± 1.07% in BON1^RSUME-KD^ tumors versus 10.15 ± 1.01% in BON1^Scramble^, *P* < 0.01) (Supplementary Figure 12), which is in line with the proliferation results *in vitro* (Supplementary Figure 11). BON1^RSUME-KD^ displayed reduced chromogranin A (CgA) immunoreactivity compared to the scramble control (Figure 7A) indicating loss of neuroendocrine differentiation. BON1^RSUME-KD^ derived tumors showed weak cytoplasmic HIF-1α immunoreactivity compared to the strong nuclear staining seen in BON1^Scramble^ (Supplementary Figure 15). In addition, BON1^RSUME-KD^ derived tumors showed slightly lower VEGF immunoreactivity (not shown) and mRNA expression compared to BON1^Scramble^ (Supplementary Figure 13B). In contrast, BON1^RSUME-KD^ tumors showed lower micro-vessel density (MVD) compared to scramble control (44.8 ± 3.6/field versus 67.4 ± 6.3/field) as determined by CD31 immunostaining (Figure 7B).

BON1^RSUME-KD^ tumors showed stronger immunoreactivity for the activated NF-κB subunit pp65/RelA-Ser536 and the NF-κB target IL-8 (Supplementary Figures 13C, 14, 16), confirming our *in vitro* data. In addition, IL-8 transcripts were elevated in the BON1^RSUME-KD^ tumors (Supplementary Figure 13C). In contrast, PTEN expression was significantly reduced in BON1^RSUME-KD^ xenografts with predominantly cytoplasmic staining (Supplementary Figures 13D, 14, 17). BON1^Scramble^ tumors showed strong expression (mostly nuclear) of PTEN, despite its pancreatic neuroendocrine tumor origin (Supplementary Figures 13D, 14, 17). Accordingly, activated pAkt-Ser473 immunoreactivity and protein levels were increased in BON1^RSUME-KD^ tumors compared to the scramble controls (Supplementary Figures 14, 18).

Five of eight (62.5%) mice injected with BON1^RSUME-KD^ cells developed liver metastasis, evidenced grossly and histologically by H&E staining (Figure 7C, 7D). In contrast, only one out of eight (12.5%) mice injected with BON1^Scramble^ cells developed liver metastasis. Markers for epithelial-mesenchymal transition (EMT), such as TGFβ, Snail, N-cadherin increased, while E-cadherin decreased in BON1^RSUME-KD^ tumors, indicating that loss of RSUME promotes EMT (Figure 7E). Altogether, our data show that loss of RSUME in PanNET cells results in PTEN loss and supports PanNET tumor and metastasis formation.

## DISCUSSION

In the present study, we demonstrate for the first time that the expression of RSUME is altered in PanNETs and present *in vitro* and *in vivo* data which strongly suggest that the reduction of RSUME contributes to the tumorigenesis and metastasis in PanNETs. RSUME is highly expressed in various endocrine/exocrine glands, including the pancreas [17]. We could show that RSUME is mainly localized in insulin-producing beta-cells and is also present in other endocrine and exocrine pancreatic cell types whereas it is not expressed in the ductal cells, indicating a distinct physiological role of RSUME in endocrine and exocrine functions of the pancreas. Whereas RSUME expression was preserved in all insulinomas studied, RSUME was mostly absent in other types of hormone-producing and in hormone-negative PanNETs. As most of the insulinomas are considered to represent well-differentiated, benign tumors we speculate that the loss of RSUME is associated with the development of more aggressive, less well-differentiated PanNETs. After this first description on the role of RSUME in PanNETs, clinical correlation studies are needed to establish the association that RSUME might have whit different types of tumors.

Tumor expansion is accompanied by transient hypoxia, which triggers tumor neovascularization. Hypoxia induces RSUME, which was found to be upregulated in the hypoxic inner zones of gliomas and pituitary adenomas [17, 32]. In breast cancer, RSUME (RWDD3) has been associated with 15 other genes as part of the risk prediction signature [33]. In a GWAS performed in 2204 breast cancer patients RWDD3 was associated with paclitaxel-induced neuropathy [34], which however could not be confirmed in a cohort of paclitaxel-treated patients with ovarian cancer [35]. Alterations of RSUME have also been associated with chronic inflammation-induced neuropathic pain [36].

Hypoxia up-regulated RSUME in the PanNET-derived BON1 cell line, but in PanNETs RSUME was down-regulated suggesting the presence of other more important regulatory mechanisms. Inflammatory processes play an important role in the development of pancreatic adenocarcinomas [2] and may be responsible for the RSUME down-regulation in PanNETs. Indeed, TNF-α which was reported to be produced intratumorally in PanNETs [36], strongly suppressed RSUME (Supplementary Figure 19). These finding are in accordance with a recent study in which chronic inflammation reduced the transcription of RWDD3/RSUME and anti-inflammatory acting drugs could revert this effect [36].

Loss of RSUME abolished the stimulatory effect of hypoxia on HIF-1α, but not on VEGF-A, suggesting the presence of an alternative compensatory mechanism. We have previously shown that RSUME inhibits NF-κB by stabilizing its inhibitor I-κBα [17]. NF-κB stimulates VEGF-A transcription directly by binding its promoter or indirectly through IL-8 expression, which acts in an autocrine fashion [21, 24]. Our data show that loss of RSUME induces IL-8 synthesis, which then triggers VEGF-A, an effect that is abolished using an IL-8 receptor CXCR2 inhibitor. Poorly differentiated PanNETs are less vascularized than well-differentiated tumors [15]. Furthermore, high expression of IL-8 and CXCR2 was observed in the human PanNET patient samples [21]. Thus, the NF-κB/IL-8 pathway may compensate the decline of HIF-1 action on VEGF-A-mediated angiogenesis in PanNET cells that have lost RSUME. This may explain why neovascularization is maintained in advanced PanNETs despite RSUME down-regulation.

Another important target, which is inhibited by NF-κB is PTEN [27]. PTEN is reduced in PanNETs and its expression is inversely correlated with survival [13, 14]. PTEN loss is rarely caused by genetic mutations in PanNETs and the mechanism remains unclear [11, 30]. In the present study, we describe for the first time that the tumor suppressor PTEN is a target of RSUME. Loss of RSUME in a PanNET model in nude mice is accompanied by a decrease in PTEN, which may be in part due to the upregulation of NF-κB that suppresses PTEN transcription [27]. In addition, recent studies demonstrated that PTEN is sumoylated [28, 38] and herein, we show that RSUME enhances PTEN sumoylation and its activity. This action on PTEN reinforces the role of RSUME on several specific targets related to cancer and inflammation, such as HIF-1α, I-κB, GR and pVHL [17, 18, 23, 32, 39]. Whether the observed loss of RSUME in the majority of human PanNETs studied is responsible for the cytoplasmatic localization or complete loss of PTEN in our series of human PanNETs needs to be confirmed in future studies.

Loss of RSUME is accompanied by decreased PTEN sumoylation and increased Akt phosphorylation, which may contribute to the PI3K pathway over-activation observed in high grade PanNETs [11, 14]. The decreased PTEN sumoylation impairs its cellular distribution. Cytosolic PTEN suppress the PI3K survival pathway, while nuclear PTEN controls DNA damage repair, genotoxic stress, chromosome stabilization and growth [29, 40, 41]. Our data show that RSUME sumoylates PTEN and increases its nuclear accumulation. Indeed, sumoylated PTEN is located in the nucleus, where it induces DNA repair upon genotoxic stress [29, 40]. Therefore, the observed RSUME down-regulation leads to loss of nuclear PTEN, which would result in impaired chromosome stabilization [40, 41].

Increased chromosomal instability renders the tumor cell susceptible to additional mutations that increase its tumorigenic and metastatic potential [2, 13, 14, 42]. Herein, we demonstrate that loss of RSUME results in high metastatic potential in orthotopic pancreatic transplants. We have chosen the orthotopic transplantation in the pancreas compared to subcutaneous transplantation, because it mimics the human PanNET condition and the metastasis formation of the tumors in the neighboring structures of the pancreas, in particular in the liver, can be monitored [30, 43]. Tumors of BON1 cells with RSUME knockdown formed multiple liver metastases that may be a consequence of increased NF-κB expression and/or loss of PTEN [12, 30, 44]. The BON1^RSUME-KD^-derived tumors were also much larger, had higher proliferation rates and signs of high-grade less well-differentiated tumors as shown by morphological H&E staining and decreased CgA expression. In addition, microvessel density was low in these tumors similar to what is observed in the human PanNETs [15].

Our data demonstrate that RSUME affects multiple targets in PanNET cells and as long as RSUME expression is preserved, these tumors show relatively low proliferation rates, expand slowly and have a limited metastatic potential despite elevated angiogenesis and enhanced microvessel density. The decline of RSUME in PanNETs goes along with reduced HIF-1α/VEGF-A, elevated NF-κB/IL-8, declined PTEN and enhanced PI3K/Akt/mTOR activation. These multiple changes may explain why mono-targeting pharmacological treatment concepts often failed in tumor therapy and therefore combined application of drugs directed against different targets gave better results [5–8]. Altogether, our findings demonstrate considerable evidence that loss of RSUME is involved in the increase of tumor aggressiveness and metastases formation in PanNETs. After our first description on the involment of RSUME in PanNET, further studies with higher numbers of PanNETs are needed in which the correlations between RSUME expression and histological characteristics of the tumors as well as clinical parameters and outcome of affected patients are investigated.

## MATERIALS AND METHODS

### Human tissue samples

Paraffin-embedded tissue slides of 9 normal pancreas tissue samples, 24 human pancreatic neuroendocrine tumors and intraoperatively removed adjacent normal human pancreatic tissue were obtained from the Department of Pathology of the Technical University Munich, Germany. The histopathological diagnosis and grading, as well as staging followed the recommendations of the WHO (Table 1). Sampling of tissues and usage of clinical data for scientific purposes was approved by the institutional ethics committee.

### Cell lines and reagents

Human pancreatic endocrine tumor BON1 cells used in the study were authenticated by Eurofins (Ebersberg, Germany). HEK293 cells and COS7 cells were obtained from American Type Culture Collection (ATCC; Manassas, VA, USA) and were cultured - like BON1 cells - at 5% CO_2_ and 37°C in DMEM (pH 7.3) supplemented with 10% FCS, 2mM glutamine, 0.5 mg/l partricin and 10^5^ U/liter penicillin-streptomycin. In addition to BON1 cells, human pancreatic endocrine carcinoma QGP-1 cells were used for several control experiments as some authors suggest that QGP1 cells were a better model for PanNETs. However, in a recent study only limited differences between the two cell types were found [45]. QGP1 cells were obtained from the Health Science Research Resources Bank (Osaka, Japan) and maintained in RPMI1640 medium supplemented with 10% FCS, 2mM glutamine, 0.5 mg/l partricin and 10^5^ U/liter penicillin-streptomycin. Cell culture materials and reagents were obtained from Life Technologies, Falcon (Heidelberg, Germany), Nunc (Wiesbaden, Germany), Seromed (Berlin, Germany), Flow Cytometry Standards Corp. (Meckenheim, Germany) and Sigma. Cobalt chloride (CoCl_2_) was purchased from Sigma. For hypoxia, cells were incubated in 2% serum at 37°C, 5% CO_2_, and 1% O_2_ balanced with N_2_ using a hypoxic chamber ProOx Model 110 (BioSpherix).

### RNA isolation and RT-PCR

Total RNA was extracted from cells and tumor tissues with Trizol reagent (Life Technology) according to the manufacture's instruction. 500 ng total RNA was reverse transcribed using Oligo-dT under standard conditions provided by the manufacture (Life technology). Quantitative real-time PCR was performed on a LightCycler (Roche) using LightCycler FastStart DNA Master SYBR Green Plus (Roche) in a final volume of 10 μl. Primer sequences and conditions for RT-PCR are listed in Supplementary Table 1. Expression levels of the housekeeping genes human β-actin were used for normalization.

### Transfection and generation of target-specific silent cells

All constructs used in this study are listed in Supplementary Table 2. Sumoylation deficient PTEN mutations (K254R, K266R, K254/266R) were generated by site-directed mutagenesis assay as described previously. Transfection assay was performed with lipofectamin 2000 following the manufacturer's instructions. The reporter constructs (HRE-Luc, RSUME-Luc, RSUME-ΔHRE-Luc, IL-8-Luc, IL-8-Δ-NFκB-Luc) were described previously. 500 ng of reporter constructs with 300 ng β-galactosidase were co-transfected into BON1 cells and luciferase activity was measured with the Dual Luciferase Reporter Assay System (Promega). The relative luciferase activity was calculated by the ratio of luciferase/ β-gal activity. For stable RSUME knockdown generation, the plasmids encoding shRSUME or scramble RNA (SABiosciences, USA) were transfected with lipofectamin 2000 into the BON1 cells according to the standard protocol. The stable clones were selected with Geneticin (Life Technologies) at a concentration of 1 mg/ml. After the selection, stable clones with RSUME knockdown (BON1^RSUME-KD^) and scramble (BON1^Scramble^) were cultured in medium with 500 μg/ml Geneticin and used at passage 4 and 5. RSUME expression level was validated by RT-PCR and western blot. All the data shown were from one individual clone designated No.15; similar results were obtained in other individual clones as well as in transient expression assays.

### Western blot and antibodies

Western blot analysis was carried out on whole cell extracts (50 μg) upon various treatments, after fractionation by PAGE gel electrophoresis and transferred to PVDF membranes for immunoblotting with antibodies listed in Supplementary Table 3. HRP-conjugated secondary antibodies against rabbit and mouse were all obtained from Cell Signalling Tech. The ECL system (Clarity ECL substrate, Biorad) and hyperfilm (GE Healthcare, Munich, Germany) were used for membrane visualization.

### ELISA

Measurement of VEGF-A and IL-8 secretion in cell culture supernatant was performed with ELISA kits (R&D Systems, Wiesbaden, Germany) for human VEGF-A and human IL-8 according to the manufacturer's instruction. All the experiments were carried out in quadruplicates.

### Immunoprecipitation

Cells for immunoprecipitation assay were lysed in Immunoprecipitation lysis buffer (25 mM Tris PH 8.0, 150 mM NaCl, 1% NP-40, 1 mM EDTA, 20 mM NEM) containing protease inhibitor cocktail (1:100, Sigma). The whole cell lysates obtained from centrifugation were first precleared by Dynabeads Protein G magnetic (Invitrogen) for 1 hour and then incubated with specific antibodies (PTEN, # 9558, Cell Signaling, 1:100) overnight at 4°C with rotation. The protein G magnetic beads were added the following day to the lysates and further incubate for 2 hours at 4°C with rotation. The immunocomplexes were then washed with Immunoprecipitation lysis buffer three times and then boiled with sample loading buffer and subjected to SDS-PAGE followed by western blot analysis.

### *In vitro* sumoylation assay

The His-tagged recombinant protein pQE30, pQE30-RSUME were transformed into E. Coli M15 (pREP4) cells with the Qiaexpress kit according to the manufacturer's instructions. GST-PTEN expressed in E.Coli DH5α and purified with glutathione-sepharose 4B beads (GE Healthcare) as specified by the manufacture. The protein concentration was measured by Bradford (Biorad) and then recombinant proteins were validated by western blot for the correct expression.

*In vitro* sumoylation conjunction assay was performed using SUMO kit from Enzo bioscience. Specifically, 250 ng GST-PTEN and mutants, 1 μl of SUMO2, 1 μl Aos1&Uba2, 1 μl Ubc9, and 2 μl 10× reaction buffer with or without 1 μl ATP; H2O was added to make the final volume of 20 μl. The reaction mixture was incubated at 37°C for 2 hours and stopped by adding sample loading buffer. The reaction mixture was separated by SDS-PAGE and subsequently immunoblotted with anti-PTEN antibody to detect SUMO modified PTEN.

### Sumoylation conjugation assay in cells

COS7 cells were transfected with either control plasmid or V5-RSUME plasmid together with His-SUMO1/2 and V5-Ubc9. 48 hours post-transfection, cells were harvested in PBS with protease inhibitor and divided by two parts. 10% of the cells were preserved as input in SDS-PAGE loading buffer and subjected to WB. The remaining cells were first centrifuged then lysed in Ni-NTA lysis buffer and subsequently subjected to protein purification by Nickel magnetic Sepharose beads overnight at 4°C. The beads were collected, washed 3 times with washing buffer and the antigen-antibody complexes were recovered by boiling in SDS-PAGE sample buffer. The input and samples were subjected to Western blot with anti-PTEN antibody.

### Ubiquitination assay in cells

COS7 cells were transfected with various plasmids as indicated in individual experiment. 48 h after transfection, cells were treated with 10 μM MG132 for 6 h, and the whole cell lysates were prepared with immunoprecipitation lysis buffer containing protease inhibitor cocktail and were subjected to Immunoprecipitation with anti-HA antibody. The immunoprecipitated HA-PTEN were released from the beads by boiling in SDS-PAGE sample buffer. The analysis of PTEN ubiquitination was carried out by immuno-blotting with anti-PTEN antibody.

### Protein half-life assay

COS7 cells were transfected with 1 μg/well of HA-PTEN (wild type and mutants) plasmids and 1 μg RSUME plasmid or control plasmid. 48 h after transfection, 100 μg/ml cycloheximide (CHX) was added to each well for various time points as indicated. Whole cell lysates were obtained and protein concentration was determined by Bradford assay followed by immunoblot with anti-HA antibody. β-actin was used as a loading control.

### Tumor implantation

Female nude mice (4–6 weeks) were used for tumor transplantation. Animal care followed institutional guidelines and experiments were approved by local animal research authorities. Mice were anaesthetized by intraperitoneal administration of Ketavel and Rompun as following the standard protocol. For tumor induction, the pancreas was exposed and 50 μl matrigel and BON1 cell mixture (1:1, 5 × 10^5^) were injected into the head of the pancreas. After 9 weeks, mice were sacrificed; primary tumors and liver were collected.

### Immunohistochemistry and immunofluorescence

For immunocytofluorescence experiments, cells or cryostat cut (5 μm) tumor tissues were fixed in 4% paraformaldehyde for 5 minutes, and then blocked in 5% goat serum with 0.1% (v/v) triton X100 for 1 hour at room temperature. Slides were incubated with the indicated antibodies overnight at 4°C and then washed and incubated with Alexa Fluor^®^ 594 goat anti-rabbit antibody (1:500, Invitrogen) or FITC-488 goat anti-mouse (Invitrogen) antibody at room temperature for 2 hours. After washing with PBS, ProLong^®^ Gold antifade reagent with DAPI (Invitrogen) was used for mounting and visualization of cell nuclei. Images were obtained using a confocal microscope (Fluo View TM FV1000, Olympus). Images were obtained using 40× objectives. The antibodies used for staining are listed in Supplementary Table 4. For human pancreatic neuroendocrine tumors as well as normal pancreas slides, paraffin embedded samples were first subjected to deparaffinization and citric acid based antigen retrieval was performed following standard protocols. Sections were either stained with hematoxylin and eosin (H&E) or subjected to immunohistochemistry or immunofluorescence. Antibodies used in this study are listed in Supplementary Table 4. Immunohistochemistry images were obtained with light microscope (Zeiss, Germany) with 20× objectives. Immunofluorescence images were obtained using confocal microscope (Fluo ViewTM FV1000, Olympus). Images were obtained using 60× objectives.

### Determination of microvessel density and Ki67 index in mouse xenografts

For quantification of microvessel density (MVD), the average number of CD31 positive vessels in a 0.6 mm^2^ (10×) measurement area was determined from 4 regions of maximal vascular density from control and RSUME knockdown tumors (*n* = 6). Ki67 index was obtained by counting the ratio of Ki67 positive cells versus total nuclei (DAPI). 4 randomly selected images were chosen from control and RSUME knockdown tumors (*n* = 6) for calculation (Imagine J, NIH).

### Statistical analysis

Each of the experiments was repeated at least three times. The individual experiments were performed in quadruplicate wells. All data are presented as mean ± SEM unless otherwise specified. For all two-way comparisons, unpaired *t*-tests were used. Statistical analysis was performed using the unpaired Student's *t*-test and were considered statistically significant if *P* < 0.05.

## SUPPLEMENTARY MATERIALS FIGURES AND TABLES



## References

[1] Kloppel G (2011). Classification and pathology of gastroenteropancreatic neuroendocrine neoplasms. Endocr Relat Cancer.

[2] Capurso G, Festa S, Valente R, Piciucchi M, Panzuto F, Jensen RT, Delle Fave G (2012). Molecular pathology and genetics of pancreatic endocrine tumours. J Mol Endocrinol.

[3] Chandra R, Liddle RA (2013). Modulation of pancreatic exocrine and endocrine secretion. Curr Opin Gastroenterol.

[4] McKenna LR, Edil BH (2014). Update on pancreatic neuroendocrine tumors. Gland Surg.

[5] Ellison TA, Edil BH (2012). The current management of pancreatic neuroendocrine tumors. Adv Surg.

[6] Auernhammer CJ, Göke B (2011). Therapeutic strategies for advanced neuroendocrine carcinomas of jejunum/ileum and pancreatic origin. Gut.

[7] Teule A, Casanovas O (2012). Relevance of angiogenesis in neuroendocrine tumors. Target Oncol.

[8] Khagi S, Saif MW (2015). Pancreatic neuroendocrine tumors: targeting the molecular basis of disease. Curr Opin Oncol.

[9] Oberg K (2013). The genetics of neuroendocrine tumors. Sem Oncol.

[10] de Wilde RF, Edil BH, Hruban RH, Maitra A (2012). Well-differentiated pancreatic neuroendocrine tumors: from genetics to therapy. Nat Rev Gastroenterol Hepatol.

[11] Jiao Y, Shi C, Edil BH, de Wilde RF, Klimstra DS, Maitra A, Schulick RD, Tang LH, Wolfgang CL, Choti MA, Velculescu VE, Diaz LA, Vogelstein B (2011). DAXX/ATRX, MEN1, and mTOR pathway genes are frequently altered in pancreatic neuroendocrine tumors. Science.

[12] Kang X, Li J, Zou Y, Yi J, Zhang H, Cao M, Yeh ET, Cheng J (2010). PIASy stimulates HIF1alpha SUMOylation and negatively regulates HIF1alpha activity in response to hypoxia. Oncogene.

[13] Krausch M, Raffel A, Anlauf M, Schott M, Willenberg H, Lehwald N, Hafner D, Cupisti K, Eisenberger CF, Knoefel WT (2011). Loss of PTEN expression in neuroendocrine pancreatic tumors. Horm Metab Res.

[14] Missiaglia E, Dalai I, Barbi S, Beghelli S, Falconi M, della Peruta M, Piemonti L, Capurso G, Di Florio A, delle Fave G, Pederzoli P, Croce CM Scarpa A (2010). Pancreatic endocrine tumors: expression profiling evidences a role for AKT-mTOR pathway. J Clin Oncol.

[15] Couvelard A, O'Toole D, Turley H, Leek R, Sauvanet A, Degott C, Ruszniewski P, Belghiti J, Harris AL, Gatter K, Pezzella F (2005). Microvascular density and hypoxia-inducible factor pathway in pancreatic endocrine tumours: negative correlation of microvascular density and VEGF expression with tumour progression. Br J Cancer.

[16] Webb JD, Coleman ML, Pugh CW (2009). Hypoxia, hypoxia-inducible factors (HIF), HIF hydroxylases and oxygen sensing. Cell Mol Life Sci.

[17] Carbia-Nagashima A, Gerez J, Perez-Castro C, Paez-Pereda M, Silberstein S, Stalla GK, Holsboer F, Arzt E (2007). RSUME, a small RWD-containing protein, enhances SUMO conjugation and stabilizes HIF-1alpha during hypoxia. Cell.

[18] Shan B, Gerez J, Haedo M, Fuertes M, Theodoropoulou M, Buchfelder M, Losa M, Stalla GK, Arzt E, Renner U (2012). RSUME is implicated in HIF-1-induced VEGF-A production in pituitary tumour cells. Endocr Relat Cancer.

[19] Antico Arciuch VG, Tedesco L, Fuertes M, Arzt E (2015). Role of RSUME in inflammation and cancer. FEBS Lett.

[20] Hoesel B, Schmid JA (2013). The complexity of NF-kappaB signaling in inflammation and cancer. Mol Cancer.

[21] Hussain F, Wang J, Ahmed R, Guest SK, Lam EW, Stamp G, El-Bahrawy M (2010). The expression of IL-8 and IL-8 receptors in pancreatic adenocarcinomas and pancreatic neuroendocrine tumours. Cytokine.

[22] Schmitt AM, Riniker F, Anlauf M, Schmid S, Soltermann A, Moch H, Heitz PU, Klöppel G, Komminoth P, Perren A (2008). Islet 1 (Isl1) expression is a reliable marker for pancreatic endocrine tumors and their metastases. Am J Surg Pathol.

[23] Druker J, LIberman AC, Antunica-Noguerol M, Gerez J, Paez-Pereda M, Rein T, Iniguez-Lluhi JA, Holsboer F, Arzt E (2013). RSUME enhances glucocorticoid receptor SUMOylation and transcriptional activity. Mol Cell Biol.

[24] Mizukami Y, Jo WS, Duerr EM, Gala M, Li J, Zhang X, Zimmer MA, Iliopoulos O, Zukerberg LR, Kohgo Y, Lynch MP, Rueda BR Chung DC (2005). Induction of interleukin-8 preserves the angiogenic response in HIF-1alpha-deficient colon cancer cells. Nat Med.

[25] Kunsch C, Rosen CA (1993). NF-kappa B subunit-specific regulation of the interleukin-8 promoter. Mol Cell Biol.

[26] Desterro JM, Rodriguez MS, Hay RT (1998). SUMO-1 modification of IkappaBalpha inhibits NF-kappaB activation. Mol Cell.

[27] Vasudevan KM, Gurumurthy S, Rangnekar VM (2004). Suppression of PTEN expression by NF-kappa B prevents apoptosis. Mol Cell Biol.

[28] Huang J, Yan J, Zhang J, Zhu S, Wang Y, Shi T, Zhu C, Chen C, Liu X, Cheng J, Mustelin T, Feng GS, Chen G (2012). SUMO1 modification of PTEN regulates tumorigenesis by controlling its association with the plasma membrane. Nat Commun.

[29] Bassi C, Ho J, Srikumar T, Dowling RJ, Gorrini C, Miller SJ, Mak TW, Neel BG, Raught B, Stambolic V (2013). Nuclear PTEN controls DNA repair and sensitivity to genotoxic stress. Science.

[30] Perren A, Komminoth P, Saremaslami P, Matter C, Feurer S, Lees JA, Heitz PU, Eng C (2000). Mutation and expression analyses reveal differential subcellular compartmentalization of PTEN in endocrine pancreatic tumors compared to normal islet cells. Am J Pathol.

[31] Scholz A, Wagner K, Welzel M, Remlinger F, Wiedenmann B, Siemeister G, Rosewicz S, Detjen KM (2009). The oral multitarget tumour growth inhibitor, ZK 304709, inhibits growth of pancreatic neuroendocrine tumours in an orthotopic mouse model. Gut.

[32] Gerez J, Fuertes M, Tedesco L, Silberstein S, Sevlever G, Paez-Pereda M, Holsboer F, Turjanski AG, Arzt E (2013). In silico structural and functional characterization of the RSUME splice variants. PLoS One.

[33] Huang CC, Tu SH, Lien HH, Jeng JY, Huang CS, Huang CJ, Lai LC, Chuang EY (2013). Concurrent gene signatures for han chinese breast cancers. PloS One.

[34] Schneider BP, Li K, Miller K, Flockhart DA, Radovich M, Hancock BA (2011). Genetic associations with taxane-induced neuropathy by a genome-wide association study (GWAS) in E5103. J Clin Oncol.

[35] Bergmann TK, Vach W, Feddersen S, Eckhoff L, Green H, Herrstedt J, Brosen K (2013). GWAS-based association between RWDD3 and TECTA variants and paclitaxel induced neuropathy could not be confirmed in Scandinavian ovarian cancer patients. Acta Oncol.

[36] Rojewska E, Korostynski M, Przewlocki R, Przewlocka B, Mika J (2014). Expression profiling of genes modulated by minocycline in a rat model of neuropathic pain. Mol Pain.

[37] Karakaxas D, Gazouli M, Coker A, Agalianos C, Papanikolaou IS, Patapis P, Liakakos T, Dervenis C (2014). Genetic polymorphisms of inflammatory response gene TNF-alpha and its influence on sporadic pancreatic neuroendocrine tumors predisposition risk. Med Oncol.

[38] Wang W, Chen Y, Wang S, Hu N, Cao Z, Wang W, Tong T, Zhang X (2014). PIASxalpha ligase enhances SUMO1 modification of PTEN protein as a SUMO E3 ligase. J Biol Chem.

[39] Gerez J, Tedesco L, Bonfiglio JJ, Fuertes M, Barontini M, Silberstein S, Wu Y, Renner U, Paez-Pereda M, Holsboer F, Stalla GK, Arzt E (2015). RSUME inhibits VHL and regulates its tumor suppressor function. Oncogene.

[40] Shen WH, Balajee AS, Wang J, Wu H, Eng C, Pandolfi PP, Yin Y (2007). Essential role for nuclear PTEN in maintaining chromosomal integrity. Cell.

[41] Denning G, Jean-Joseph B, Prince C, Durden DL, Vogt PK (2007). A short N-terminal sequence of PTEN controls cytoplasmic localization and is required for suppression of cell growth. Oncogene.

[42] Baker SJ (2007). PTEN enters the nuclear age. Cell.

[43] Fraedrich K, Schrader J, Ittrich H, Keller G, Gontarewicz A, Matzat V, Kromminga A, Pace A, Moll J, Bläker M, Lohse AW, Hörsch D, Brümmendorf TH (2012). Targeting aurora kinases with danusertib (PHA-739358) inhibits growth of liver metastases from gastroenteropancreatic neuroendocrine tumors in an orthotopic xenograft model. Clin Cancer Res.

[44] Fujioka S, Sciabas GM, Schmidt C, Frederick WA, Dong QG, Abbruzzese JL, Evans DB, Baker C, Chiao PJ (2003). Function of nuclear factor kappaB in pancreatic cancer metastasis. Clin Cancer Res.

[45] Vandamme T, Peeters M, Dogan F, Pauwels P, Van Assche E, Beyens M, Mortier G, Vandeweyer G, de Herter W, Van Camp G, Hofland LJ, Op de Beeck K (2015). Whole-exome characterization of pancreatic neuroendocrine tumor cell lines BON-1 and QGP-1. J Mol Endocrinol.

