# Supplementary material for: RSUME is implicated in tumorigenesis and metastasis of pancreatic neuroendocrine tumors
Source: Oncotarget. 2016 Aug 5;7(36):57878–93. doi: 10.18632/oncotarget.11081 (PMC5295397; doi:10.18632/oncotarget.11081)
Supplement: Supplementary file 1 [file oncotarget-07-57878-s001.pdf]

RSUME is implicated in tumorigenesis and metastasis of pancreatic neuroendocrine tumors

Supplementary Materials

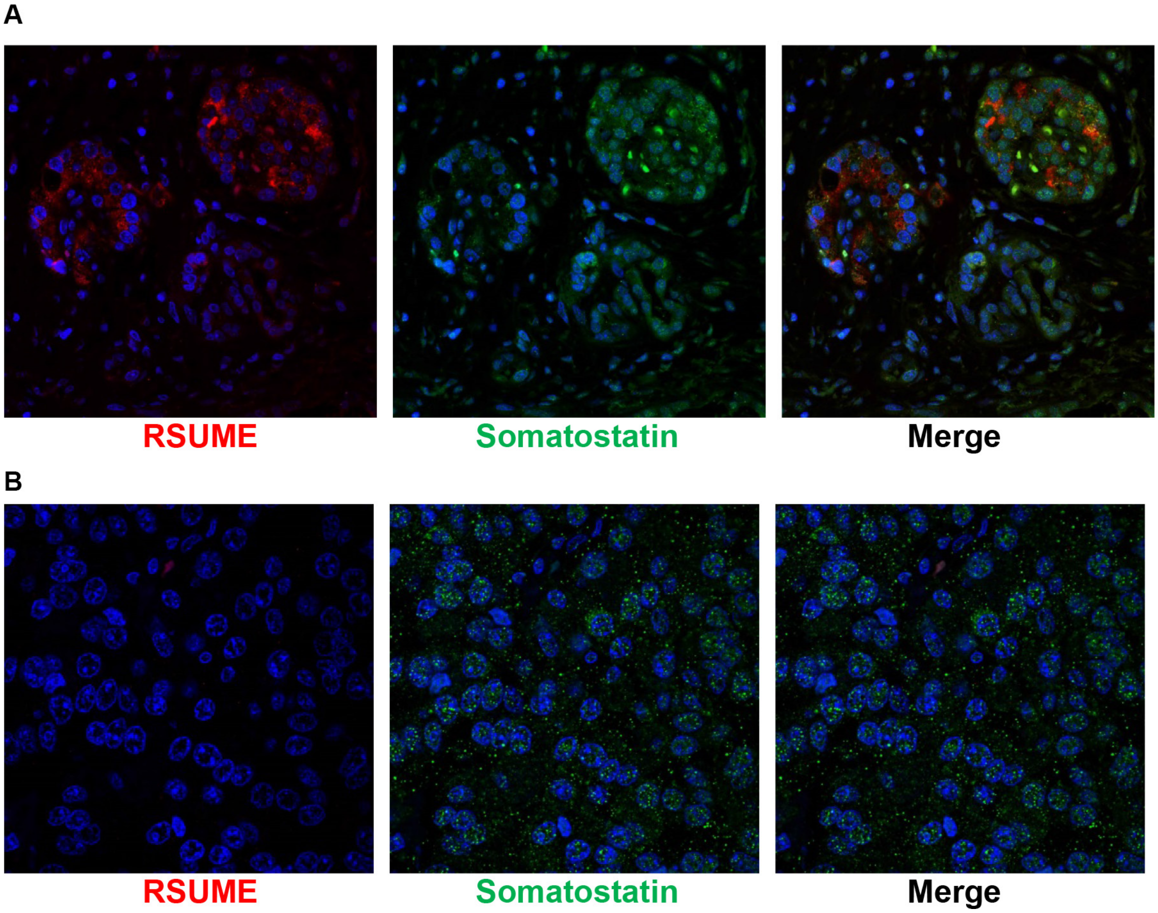

Supplementary Figure S1: Expression of RSUME (red) and somatostatin (green) in normal human pancreas (A) and in a somatostatin-positive human PanNET (B). Co-localization of RSUME and somatostatin in normal pancreas is indicated by orange color. Cell nuclei are visualized in blue (DAPI staining).

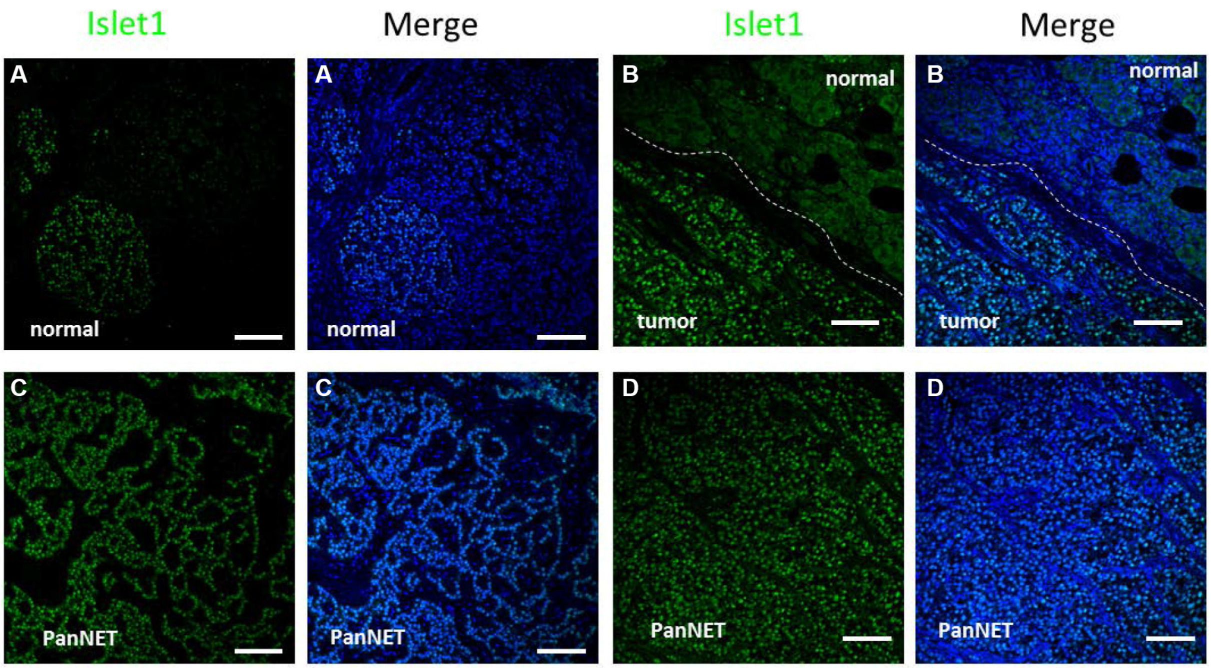

Supplementary Figure S2: Islet 1 expression in normal pancreas and pancreatic neuroendocrine tumors (PanNETs). (**A**) Islet1 displayed moderate staining pattern within pancreas Islet cells. (**B**) Islet1 shows differential staining pattern in PanNETs. In non-tumor tissue, Islet1 staining was negative, while in the malignant tumor part, Islet1 displayed strong ubiquitous staining. Islet1 displayed strong ubiquitous staining in different stage of PanNETs (C, G1; D, G2). Scale bar: 100 µm.

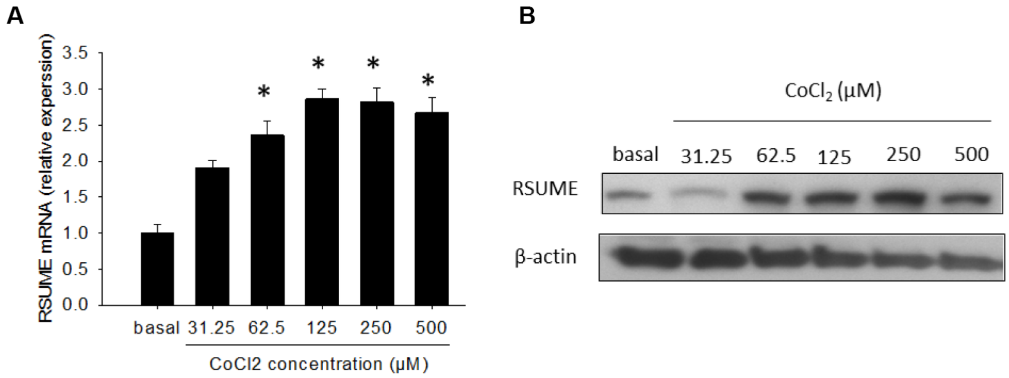

Supplementary Figure S3: RSUME is induced in hypoxia-mimicking condition in neuroendocrine tumor cells. (**A**) RSUME mRNA levels were analyzed by RT-PCR during hypoxia mimicking condition ($CoCl_2$ treatment) for indicated dose for 3 hours in pancreatic BON1 cells. β-actin was used as loading control. (**B**) RSUME protein level was determined by western blot during hypoxia mimicking conditions for indicated dose for 6 hours. Each image is representative of three independent experiments with similar results. *$P < 0.05$ vs. basal.

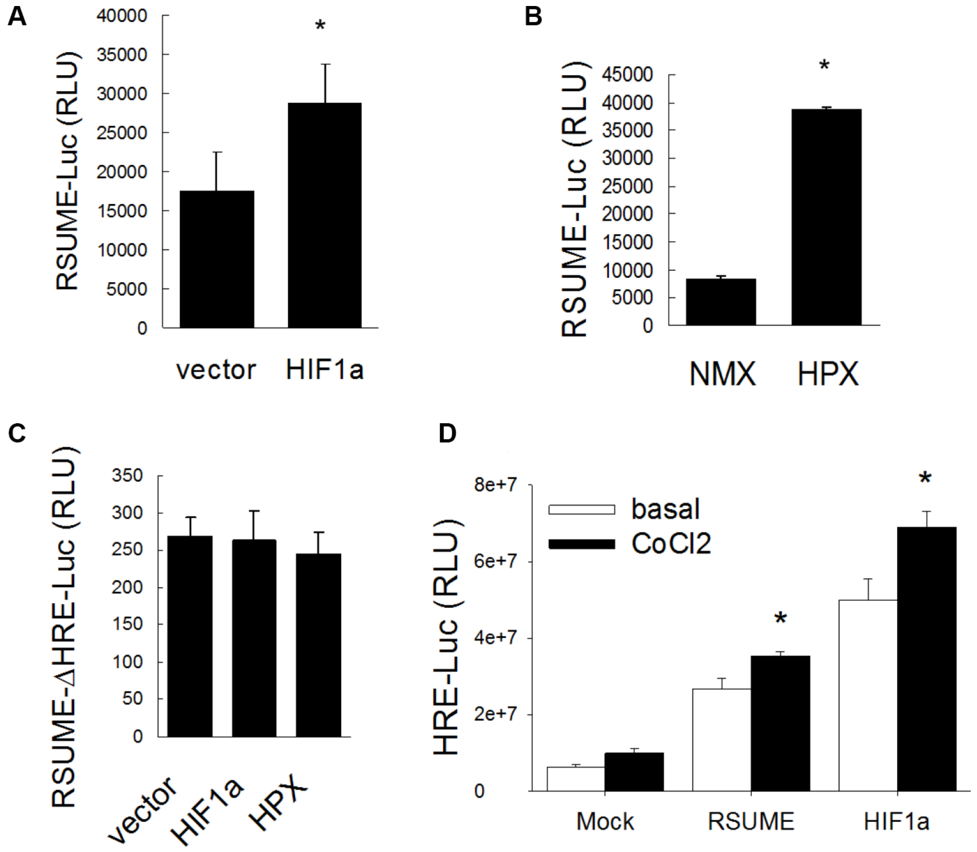

Supplementary Figure S4: RSUME/HIF-1α interaction in cells. (**A**–**C**) COS7 cells were transfected with RSUME-Luc or RSUME-ΔHRE-Luc reporter vector, together with HIF-1α, β-galactosidase (β-gal) plasmid. 24 hours post transfection, cells were subjected to hypoxic conditions for 16 hours and luciferase (LUC) activity was measured in the cell extracts. Each value was normalized to the β-gal activity (mean ± SEM of 3 different experiments, *t* test) (**D**) BON1 cells were transfected with hypoxia-response-element luciferase reporter (HRE-Luc) vector, HIF-1α or RSUME expression vector. 24 hours post transfection, cells were subjected to hypoxia-mimicking conditions for 4 hours and Luc activity was measured in cell extracts. Quantitative Real Time PCR was used to compare the mRNA level of RSUME. $^*P < 0.05$ vs. vector (A), NMX (B) or basal (D), respectively.

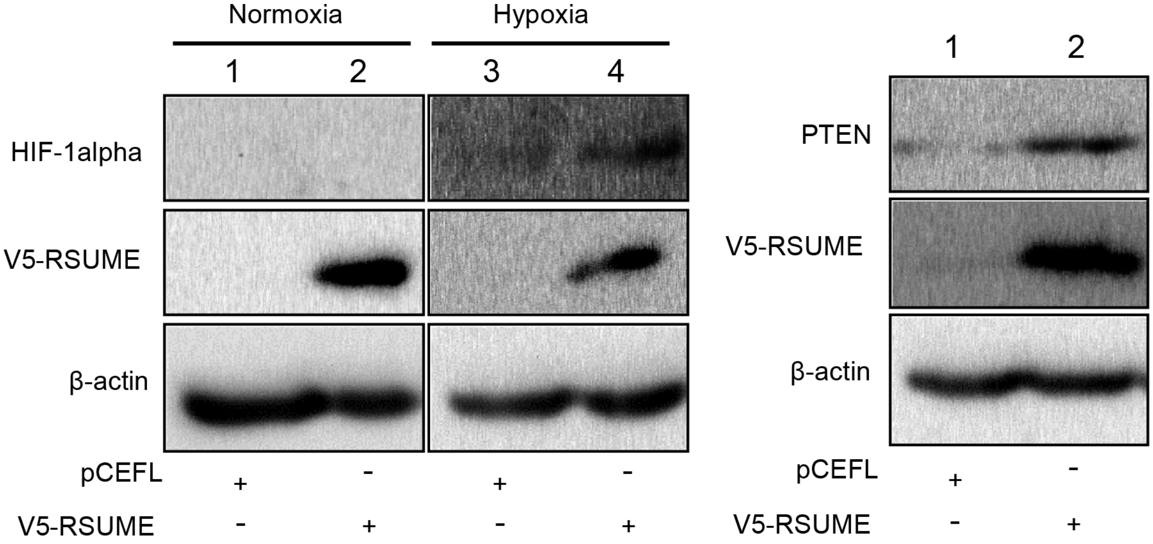

Supplementary Figure S5: Effect of RSUME overexpression in QGP1 cells on HIF-1α and PTEN. Overexpression of RSUME in neuroendocrine pancreatic QGP1 cells strongly stimulates HIF-1α expression in hypoxic QGP1 cells (left panel). Furthermore, PTEN expression is strongly enhanced in RSUME overexpressing QGP1 cells (right panel).

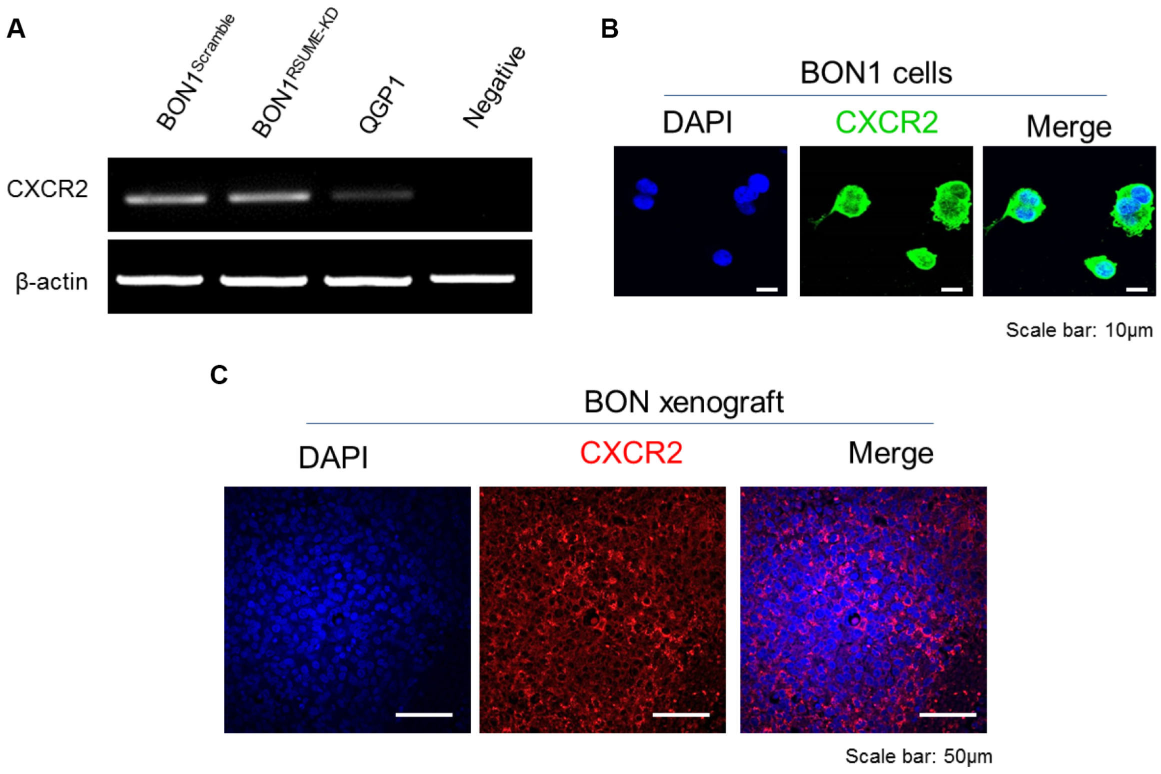

Supplementary Figure S6: CXCR2 expression in pancreatic neuroendocrine tumor cells. (**A**) IL-8 receptor CXCR2 mRNA was analyzed by semi-quantitative RT-PCR in pancreatic neuroendocrine $BON1^{Scramble}$, $BON1^{RSUME\text{-}KD}$ and QGP1 cells. β-actin was used as loading control. Immunostaining of CXCR2 was performed in BON1 cells (**B**) as well as in BON1 xenograft tumors (**C**). DAPI was used to visualize cell nuclei.

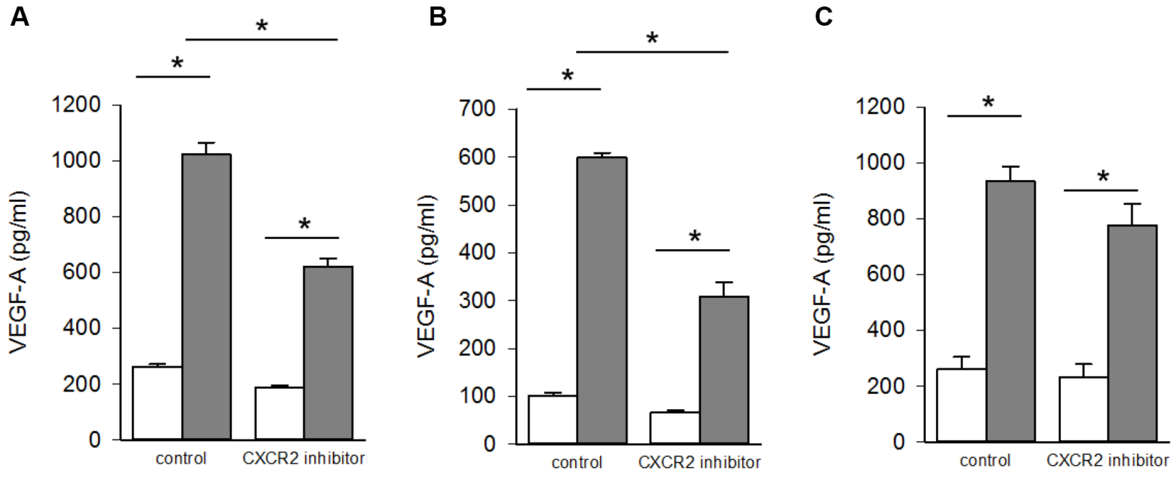

		scramble	RSUME-KD	QGP1
After CXCR2 inhibitor treatment	NMX	70%	64.5%	88.96%
	HPX	60.45%	51.6%	82.9%

Supplementary Figure S7: Influence of CXCR2 inhibition on VEGF-A secretion in PanNETs cells. Human VEGF-A secretion was measured by ELISA during hypoxia with L8 receptor CXCR2 inhibitor treatment in $BON1^{Scramble}$ (**A**), $BON1^{RSUME\text{-}KD}$ (**B**) and QGP1 cells (**C**). The values below show the percentage of VEGF-A secretion preserved when pre-treated with CXCR2 for 30 mins. White bar: normoxia; Dark grey bar: hypoxia. Results are expressed as mean ± SEM of triplicates of one representative experiment of three experiments with similar results. *$P < 0.05$.

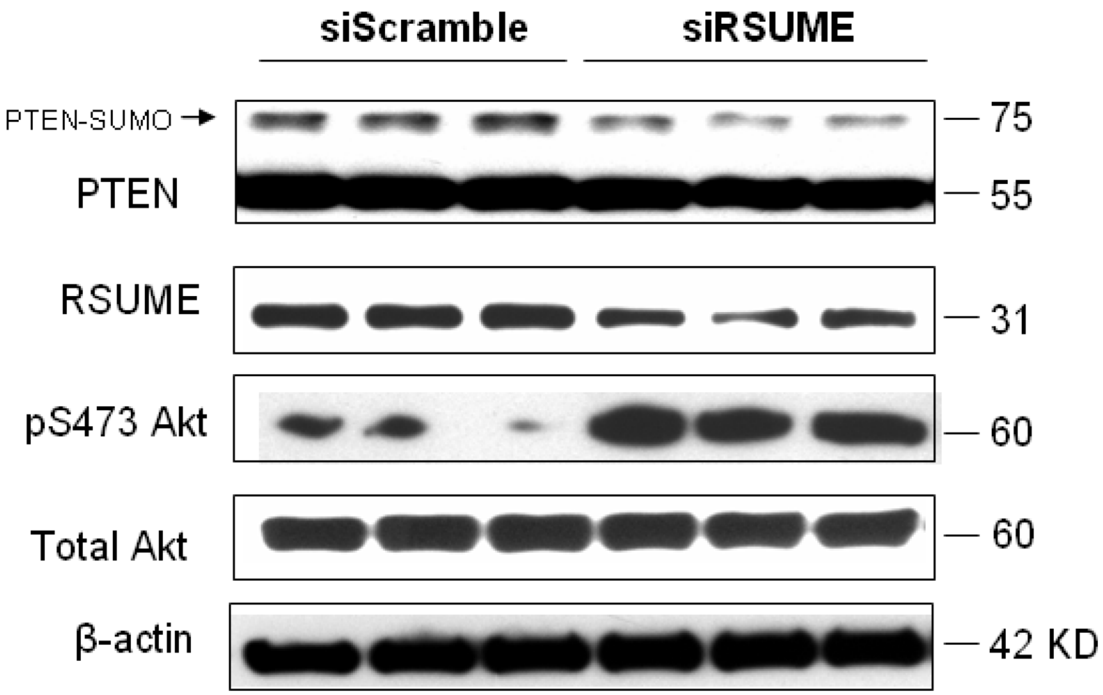

Supplementary Figure S8: RSUME knockdown led to a decrease of PTEN sumoylation and an increase of Akt phosphorylation. Human RSUME was transiently silenced by siRNA in BON1 cells. 48 hours post transfection, cell lysates were immunoblotted with antibodies against RSUME, PTEN, total Akt and phosphor-Akt (S473). β-actin was used as the loading control.

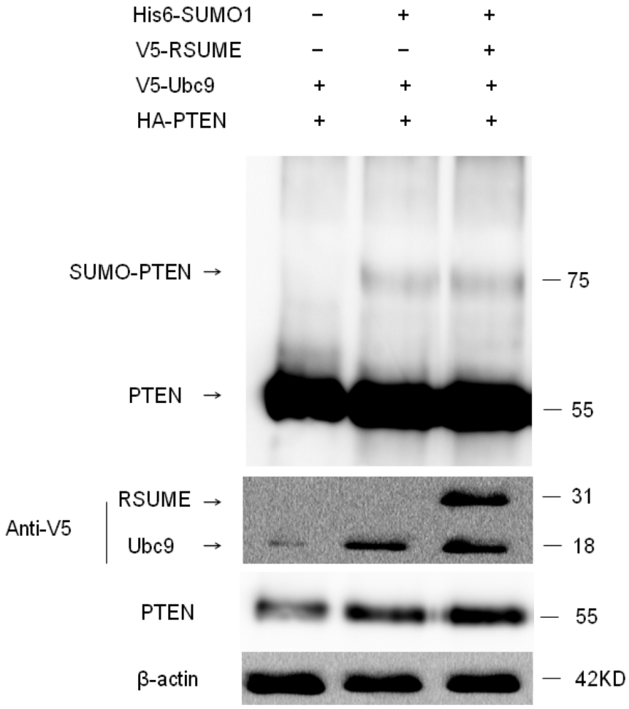

Supplementary Figure S9: Sumoylation conjugation assay in cells for SUMO1. COS7 cells were cotransfected with 0.7 µg of HA-PTEN expression plasmid, 0.5 µg wild type V5-RSUME, 0.7 µg with/without His-SUMO1 and 0.2 µg V5-Ubc9. 48 hours post transfection, cells were lysed, purified by Ni-NTA and immunoblotted with indicated antibodies.

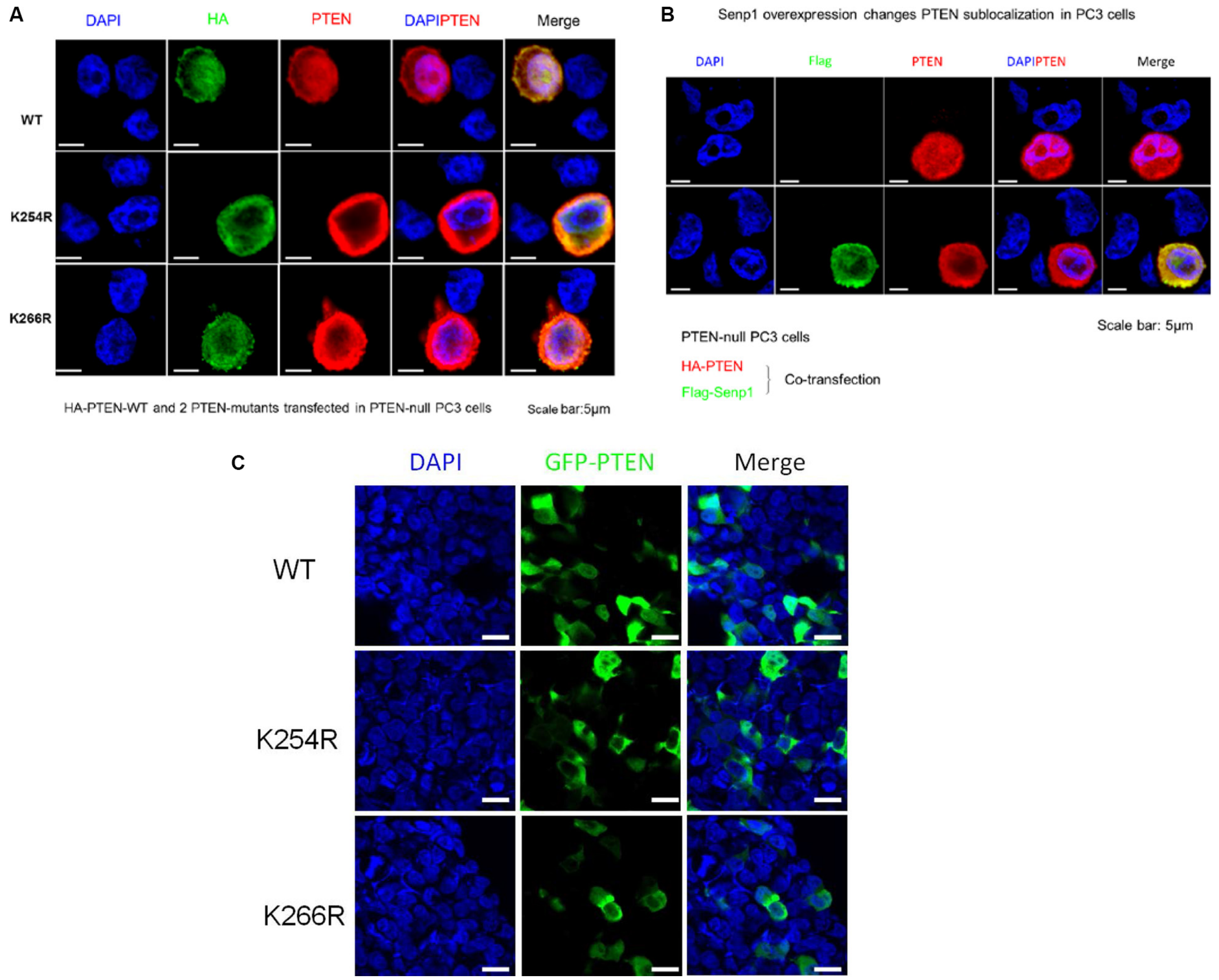

Supplementary Figure S10: PTEN sumoylation and sublocalization. (**A**) HA-PTEN and different sumoylation deficient mutants (K254R, K266R) were transfected into PTEN-null PC3 cells. 48 h after transfection, cells were fixed and stained with HA (green) and PTEN (red). Each image is representative of three independent experiments with similar results. (**B**) HA-PTEN and Flag-SENP1 was co-transfected into PTEN-null PC3 cells. 48 h after transfection, cells were fixed and stained with Flag (green) and PTEN (red). (**C**) GFP-PTEN (WT, K254R and K266R) was transfected into HEK293 cells. 48 h after transfection, cells were fixed and visualized under confocal microscope. Scale bar: 10 μm.

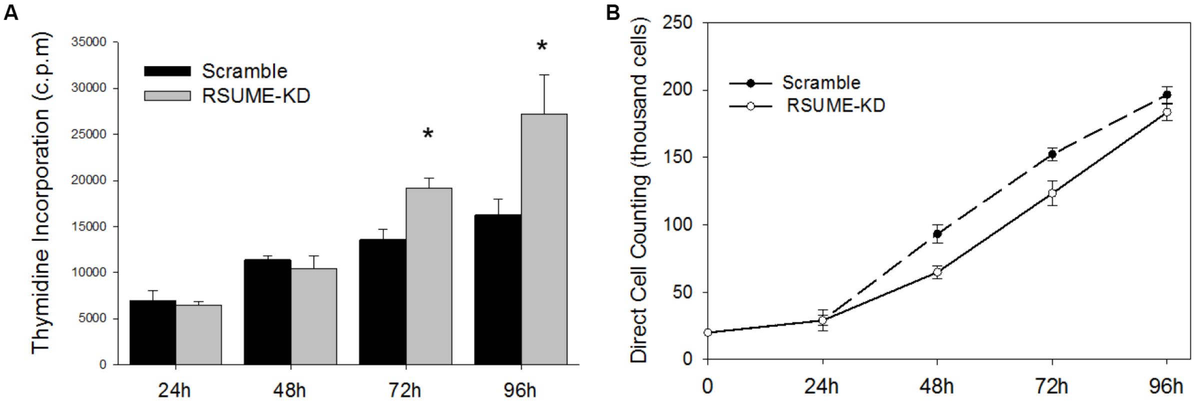

Supplementary Figure S11: ***In vitro*** **proliferation assay for** $\mathbf{BON1^{Scramble}}$ **and** $\mathbf{BON1^{RSUME\text{-}KD}}$ **cells.** (**A**) Thymidine incorporation assay was performed in $BON1^{Scramble}$ and $BON1^{RSUME\text{-}KD}$ cells. (**B**) Direct cell counting was performed in $BON1^{Scramble}$ and $BON1^{RSUME\text{-}KD}$ cells. Results are expressed as mean ± SEM of triplicates of one representative experiment of three experiments with similar results. $^{*}P < 0.05$ vs. scramble.

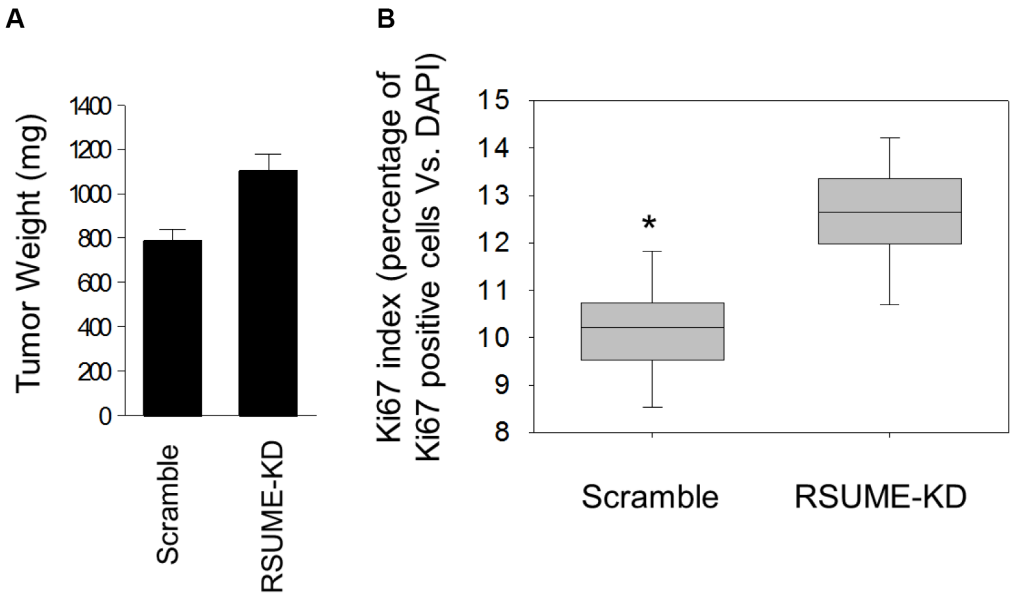

Supplementary Figure S12: *In vivo* orthotropic pancreatic tumor mouse model. 5×10^5 cells of BON1^{Scramble} or BON1$^{RSUME\text{-}KD}$ was injected into the head of the pancreas in athymic nude mice. 9 weeks later, mice were sacrificed and the pancreatic xenograft tumors as well as the livers were collected. (**A**) Orthotopic BON1 tumor weight was measured in Scramble and RSUME-KD mice ($n = 8$). (**B**) Ki67 index was calculated by the percentage of Ki67 positive cells versus DAPI (total nuclei). *$P < 0.05$ vs. RSUME-KD.

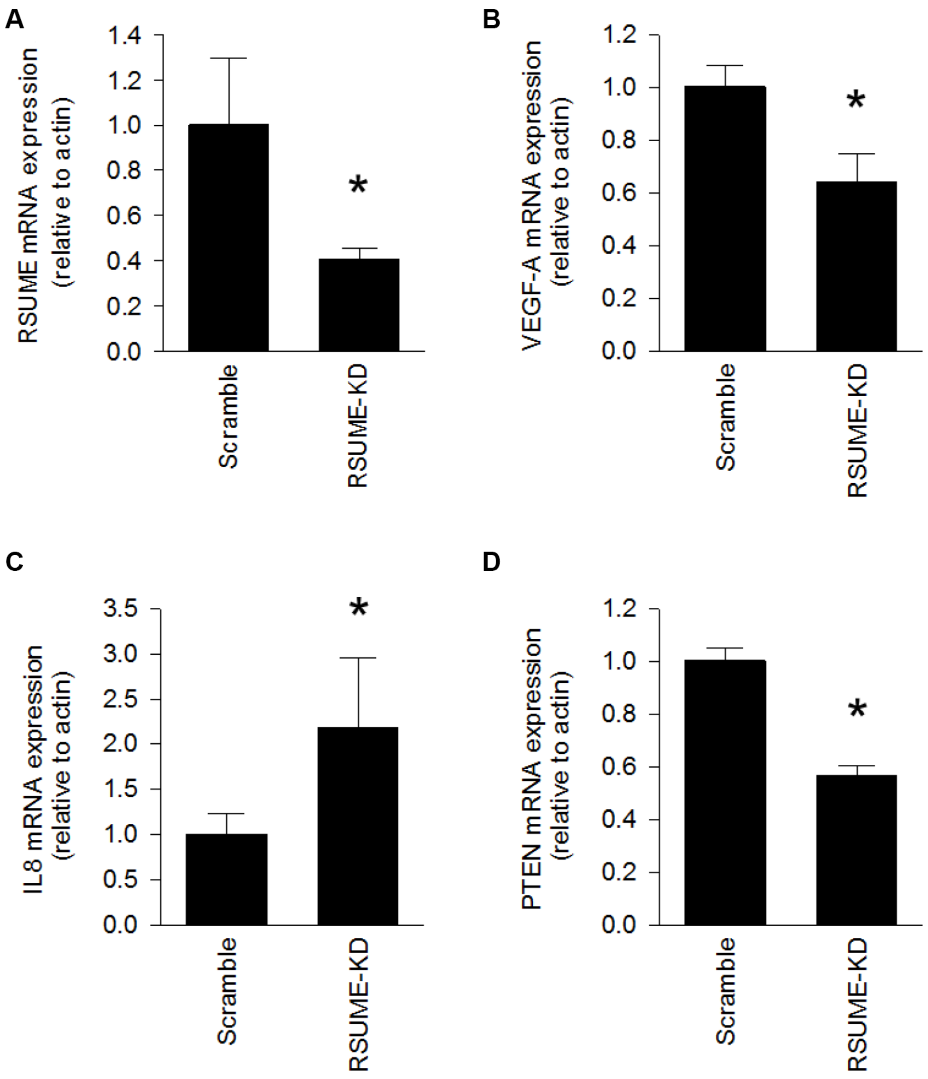

Supplementary Figure S13: Gene expression analysis of various genes from orthotopic xenograft tumors. mRNA expression of RSUME (**A**), VEGF-A (**B**), IL-8 (**C**) and PTEN (**D**) were analyzed by RT-PCR using cDNA reverse transcripts derived from BON1 cells (scramble and RSUME-KD) injected tumors. β-actin was used as loading control. *$P < 0.05$ vs scramble.

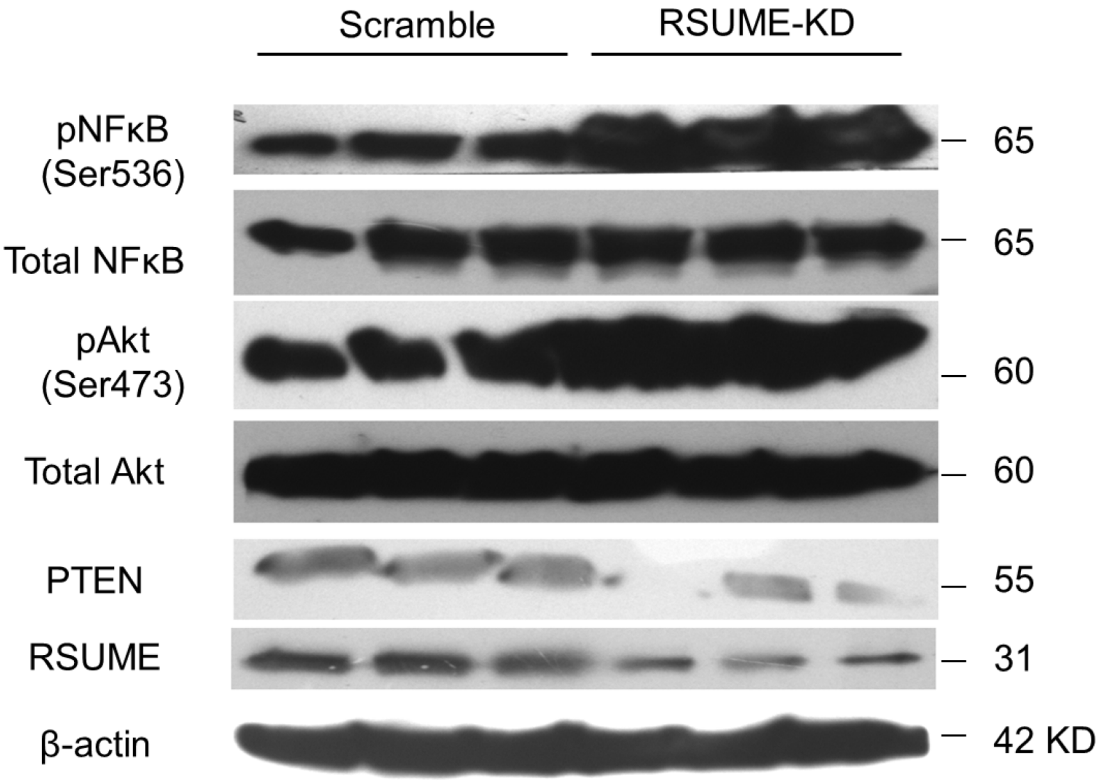

Supplementary Figure S14: Protein expression in orthotopic xenograft tumors. In protein extracts from xenograft tumors (BON1^{Scramble} and BON1$^{RSUME\text{-}KD}$) the expression of RSUME and PTEN as well as phosphorylated or total NF-κB and Akt, respectively, were analyzed with corresponding antibodies.

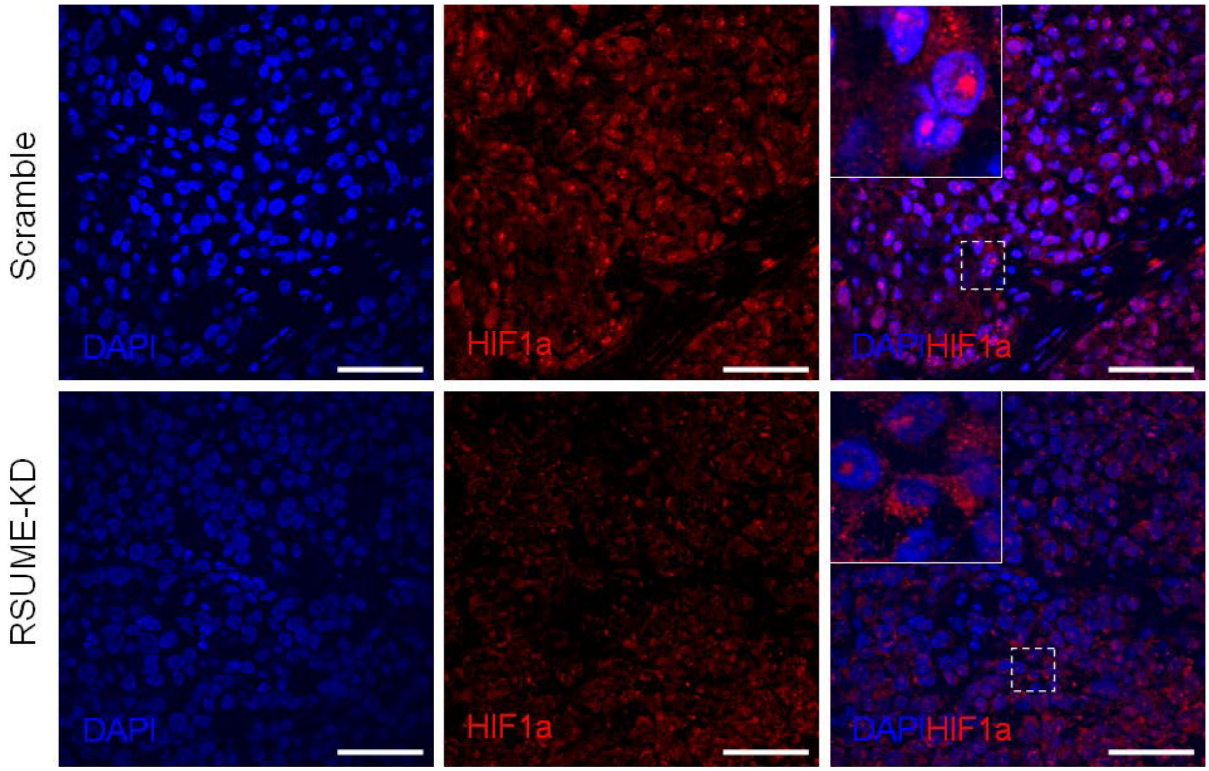

Supplementary Figure S15: Immunofluorescence staining of HIF-1α in orthotopic xenograft tumors. Immunofluorescence staining of HIF-1α (red) and DAPI (blue) in tumor orthotopic xenografts from Scramble control (scramble, upper) and RSUME knockdown (RSUME-KD, lower) mice. Scale bar: 50 µm.

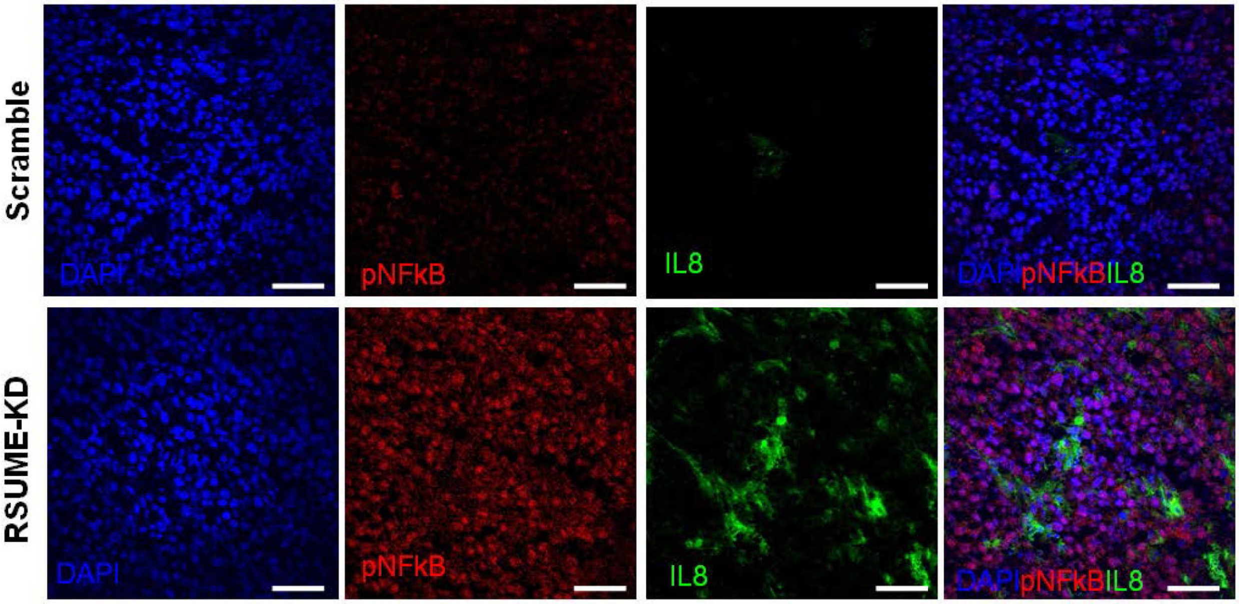

Supplementary Figure S16: Immunofluorescence staining of pNF-κB and its target IL-8 in orthotopic xenograft tumors. Immunofluorescence staining of phospho-NF-κB (Serine 536, red), IL-8 (green) and DAPI (blue) in tumor orthotopic xenografts from Scramble control (scramble, upper) and RSUME knockdown (RSUME-KD, lower) mice. Scale bar: 50 μm.

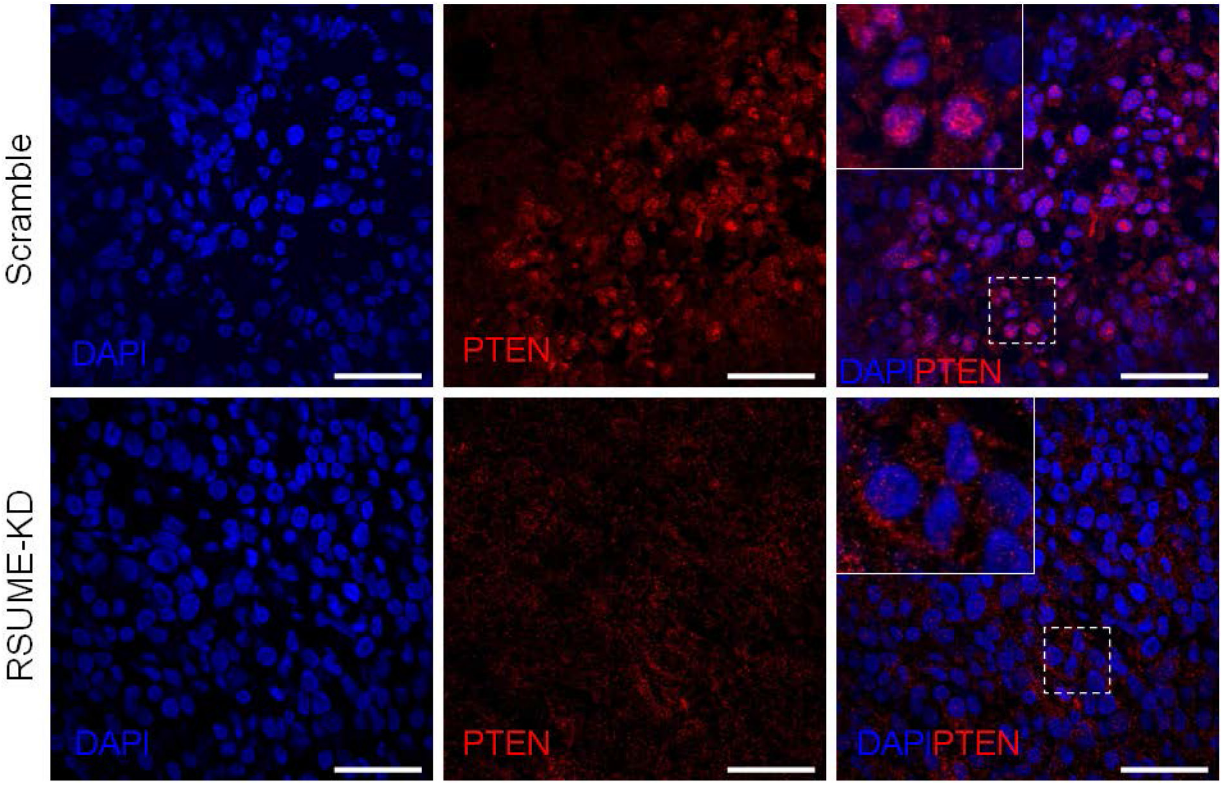

Supplementary Figure S17: Immunofluorescence staining of PTEN in orthotopic xenograft tumors. Immunofluorescence staining of PTEN (red) and DAPI (blue) in tumor orthotopic xenografts from Scramble control (scramble, upper) and RSUME knockdown (RSUME-KD, lower) mice. Scale bar: 50 μm.

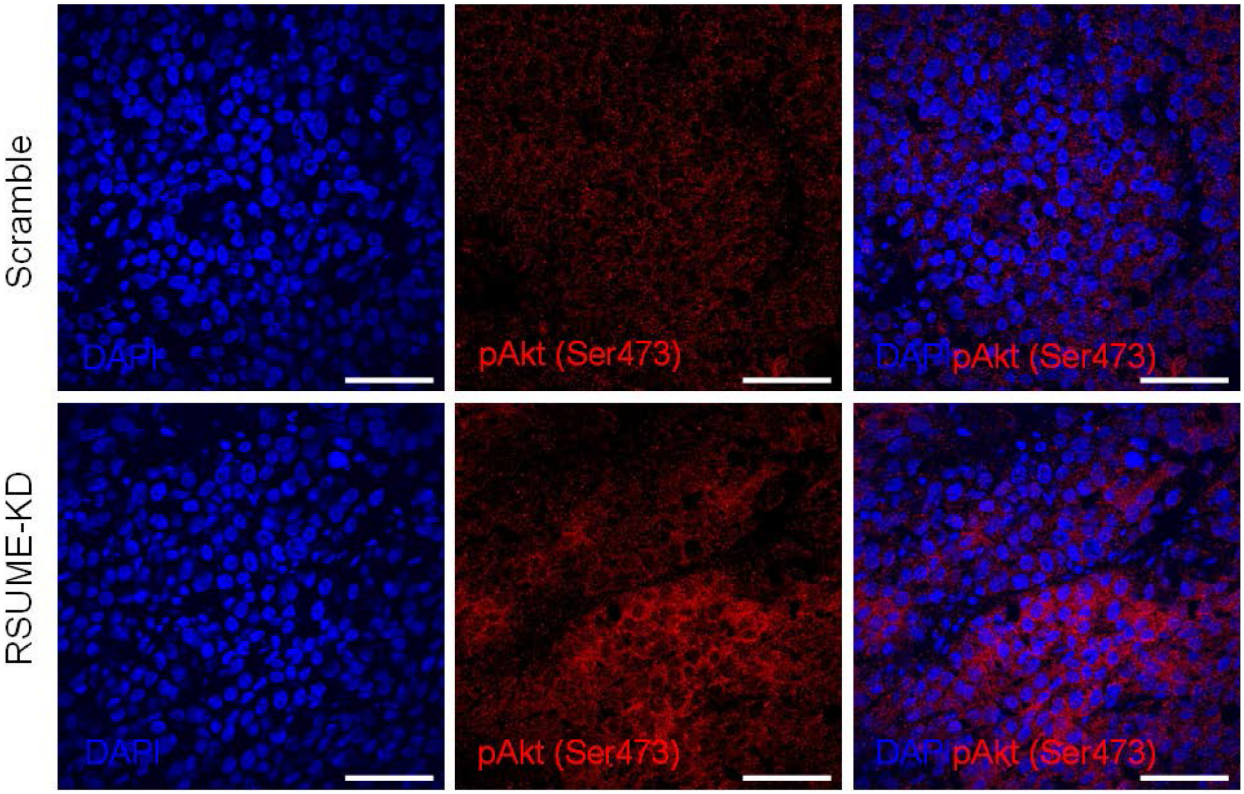

Supplementary Figure S18: Immunofluorescence staining of pAkt (Ser 473) in orthotopic xenograft tumors. Immunofluorescence staining of phosphor-Akt (Serine 473, red) and DAPI (blue) was performed in tumor orthotopic xenografts from Scramble control (scramble, upper) and RSUME knockdown (RSUME-KD, lower) mice. Scale bar: 50 µm.

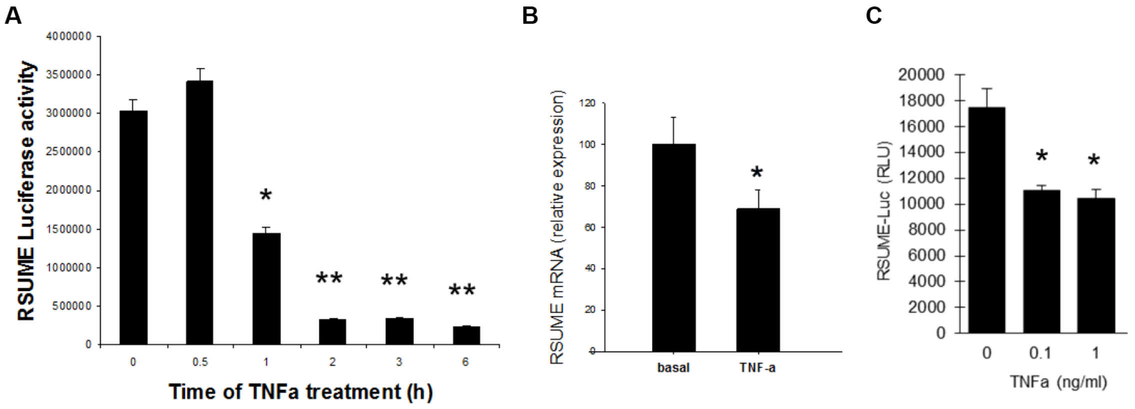

Supplementary Figure S19: The inflammatory cytokine TNF-α decreases RSUME expression. (**A**) RSUME reporter activity in BON1 cells was decreased upon TNF-α treatment with time points indicated in the graph. (**B**) RSUME mRNA expression was measured by quantitative RT-PCR under TNF-α treatment in BON1 cells. (**C**) RSUME reporter activity was measure in COS7 cells when cells were treated for 6 hours with doses indicated in the graph. *$P < 0.05$, **$P < 0.01$ vs. basal.

Supplementary Table S1: Primers for RT-PCR experiments

Oligo name	Sequence	Product size (bp)
hRSUME	F, 5′-TAC CTG GTA TCT CGA TTA ACT CTG AAC-3′<br>R, 5′-TCA GTA TTA TTT TAC CCA TGA ACA TCA-3′	300
hHIF-1α	F, 5′-CAT AGA ACA GAC AGA AAA ATC TCA TCC-3′<br>R, 5′-TTA ACT TGA TCC AAA GCT CTG AGT AAT-3′	450
hVEGF-A	F, 5′-CAG ATT ATG CGG ATC AAA CCT-3′<br>R, 5′-CAA ATG CTT TCT CCG CTC TGA-3′	148
hIL-8	F, 5′-ACA TAC TCC AAA CCT TTC CAC CC-3′<br>R, 5′-CAA CCC TCT GCA CCC AGT TTT C-3′	151
hCXCR2	F, 5′-TTT CTG GGC ATC CTT CAC AG-3′<br>R, 5′-TGC ACT TAG GCA GGA GGT CT-3′	202
hPTEN	F, 5′-ACC AGG ACC AGA GGA AAC CT-3′<br>R, 5′-GCT AGC CTC TGG ATT TGA CG-3′	241
β-actin (h)	F, 5′-ACG GGG TCA CCC ACA CTG TGC-3′<br>R, 5′-CTA GAA GCA TTT GCG GTG GAC GAT G-3′	660

Supplementary Table S2: Constructs used in the current study

Vector	Usage	Source
RSUME-Luc	luciferase reporter	from other lab
V5-RSUME	overexpression	from other lab
RSUME (ΔHRE)-Luc	luciferase reporter	from other lab
V5-RSUME (Y61A,P62A)-Mut	overexpression, sumoylation assay	from other lab
Hypoxia-responsive-element (HRE)-Luc	luciferase reporter	from other lab
Flag-HIF-1a	overexpression	from other lab
IL-8-Luc	luciferase reporter	from other lab
IL-8 (ΔNFκB)-Luc	luciferase reporter	from other lab
NF-κB-Luc	luciferase reporter	from other lab
I-κBα expression	overexpression, sumoylation assay	from other lab
I-κBα (K21,22R) Mut	overexpression, sumoylation assay	from other lab
I-κBα-super repressor	overexpression	from other lab
HA-SUMO1	sumoylation assay, ICC	from other lab
Flag-SENP1	sumoylation assay, ICC	from other lab
V5-Ubc9	sumoylation assay	from other lab
His6-SUMO1	sumoylation assay	from other lab
His6-SUMO2	sumoylation assay	from other lab
HA-PTEN	overexpression, sumoylation assay, protein degradation assay, ICC	Addgene
HA-PTEN (K254R)	sumoylation assay, ubiquitination assay, ICC	Own construct
HA-PTEN (K266R)	sumoylation assay, ubiquitination assay, ICC	Own construct
HA-PTEN (K254/266R)	sumoylation assay, ubiquitination assay, ICC	Own construct
GST-PTEN	recombinant protein purification	Addgene
GFP-PTEN	ICC	Addgene
GFP-PTEN (K254R)	ICC	Own construct
GFP-PTEN (K266R)	ICC	Own construct
GFP-PTEN (K254/266R)	ICC	Own construct
His6-Ubiqutin	ubiquitination assay	from other lab

Supplementary Table S3: Antibodies used for western blot

Antibody	Host	Company	Catalogue number	Dilution
RSUME	Rabbit	Abcam	Ab128285	1:1,000
HIF-1α	Rabbit	Cell signaling	#3716	1:1,000
NF-κB-P65	Rabbit	Santa Cruz	Sc-109	1:1,000
pP65 (Ser635)	Rabbit	Santa Cruz	Sc-33020	1:1,000
I-κBα	Rabbit	Abcam	Ab7217	1:5,000
p-I-κBα (S32+S36)	Mouse	Abcam	Ab12135	1:1,000
PTEN	Rabbit	Cell signaling	#9552	1:5,000
SUMO2	Rabbit	Enzo Life science	BML-PW9465	1:1,000
Total Akt	Rabbit	Cell signaling	#4691	1:5,000
pAkt (Ser473)	Rabbit	Cell signaling	#9271	1:750
β-actin	Mouse	R&D systems	MAB 1501	1:10,000
HA	Mouse	Thermo scientific	#26183	1:5,000
V5	Mouse	Life technology	R960-25	1:5,000
Snail	Rabbit	Cell signaling	#3879	1:1,000
TGFβ	Rabbit	Cell signaling	#3709	1:1,000
E-cadherin	Rabbit	Cell signaling	#3195	1:5,000
N-cadherin	Rabbit	Cell signaling	#13116	1:2,500

Supplementary Table S4: Antibodies used for immunohistochemistry (IHC) and immunoflorence (IF)

Antibody	Host	Company	Catalogue number	Usage	dilution
RSUME	Rabbit	Abcam	Ab128285	IHC, IF	1:100
Insulin	Mouse	Sigma	I2018	IF	1:500
Somatostatin	Goat	Santa Cruz	Sc-7819	IF	1:400
Islet1	Mouse	Abcam	Ab109517	IF	1:500
HIF-1α	Rabbit	Cell signaling	#3716	IF	1:100
VEGF-A	Rabbit	Abcam	Ab52917	IF	1:200
NFκB-P65	Rabbit	Santa Cruz	Sc-109	IF	1:100
pP65 (Ser635)	Rabbit	Santa Cruz	Sc-33020	IF	1:100
IL-8	Rabbit	Biomol	#AP8612B	IF	1:100
PTEN	Rabbit	Cell Signaling	#9559	IHC, IF	1:100
pAkt (Ser473)	Rabbit	Cell Signaling	#9271	IF	1:100
HA	Mouse	Thermo scientific	#26183	IF	1:500
V5	Mouse	Life technology	R960-25	IF	1:500
chromogranin a	Rabbit	Biomol	E1520	IHC	1:500
CD31	Goat	R & D systems	AF3628	IHC	1:500
Ki67	Mouse	BD transduction Lab	#610968	IF	1:500

Supplementary Table S5: Primers for constructs mutagenesis

Oligo name	Sequence
PTEN-K254R	F, 5′-GTG GTG ATA TCC GAG TAG AGT TCT TCC–3′<br>R, 5′-GGA AGA ACT CTA CTC GGA TAT CAC CAC–3′
PTEN-K266R	F, 5′-GAA CAA GAT GCT ACG AAA GGA CAA AAT G–3′<br>F, 5′-C ATT TTG TCC TTT CGT AGC ATC TTG TTC–3′
